# Antibodies to variable surface antigens induce antigenic variation in the intestinal parasite *Giardia lamblia*

**DOI:** 10.1038/s41467-023-38317-8

**Published:** 2023-05-03

**Authors:** Albano H. Tenaglia, Lucas A. Luján, Diego N. Ríos, Cecilia R. Molina, Victor Midlej, Paula A. Iribarren, María A. Berazategui, Alessandro Torri, Alicia Saura, Damián O. Peralta, Macarena Rodríguez-Walker, Elmer A. Fernández, Juan P. Petiti, Marianela C. Serradell, Pablo R. Gargantini, Tim Sparwasser, Vanina E. Alvarez, Wanderley de Souza, Hugo D. Luján

**Affiliations:** 1 Centro de Investigación y Desarrollo en Inmunología y Enfermedades Infecciosas (CIDIE), Consejo Nacional de Investigaciones Científicas y Técnicas (CONICET)/Universidad Católica de Córdoba (UCC), X5016HDK Córdoba, Argentina; 2 Instituto de Biofísica Carlos Chagas Filho and Centro Nacional de Biologia Estrutural e Bioimagem (CENABIO), Universidade Federal do Rio de Janeiro (UFRJ), 21941-170 Rio de Janeiro, Brazil; 3 Instituto Oswaldo Cruz, Fundação Oswaldo Cruz (FIOCRUZ), 21040-900 Rio de Janeiro, Brazil; 4 Instituto de Investigaciones Biotecnológicas (IIBIO), Consejo Nacional de Investigaciones Científicas y Técnicas (CONICET)/Universidad Nacional de General San Martín (UNSAM), B1650HMP Buenos Aires, Argentina; 5 Instituto de Investigaciones en Ciencias de la Salud (INICSA), Centro de Microscopía Electrónica, Facultad de Ciencias Médicas. CONICET/Universidad Nacional de Córdoba, X5016HUA Córdoba, Argentina; 6 Laboratorio de Parasitología y Micología, Departamento de Bioquímica Clínica, Facultad de Ciencias Químicas, Universidad Nacional de Córdoba, X5016HUA Córdoba, Argentina; 7 Institute of Medical Microbiology and Hygiene, University Medical Center of the Johannes Gutenberg-University Mainz, Mainz, Germany; 8 Present Address: Centro de Investigaciones en Química Biológica de Córdoba (CIQUIBIC), CONICET, Universidad Nacional de Córdoba, Córdoba, Argentina; 9 Present Address: Clínica Universitaria Reina Fabiola, Universidad Católica de Córdoba, Córdoba, Argentina; 10 Present Address: Department of Life Sciences, Sir Alexander Fleming Building, Imperial College London, London, UK; 11 Present Address: Viruses and RNA Interference Unit, CNRS Unité Mixte de Recherche, Institut Pasteur, Paris, France; 12 Present Address: Cátedra de Química Biológica, Facultad de Ciencias de la Salud, Universidad Nacional de Córdoba, Córdoba, Argentina; 13 Present Address: Fundación para el progreso de la Medicina, Córdoba, Argentina

**Keywords:** Parasite immune evasion, Parasite biology

## Abstract

The genomes of most protozoa encode families of variant surface antigens. In some parasitic microorganisms, it has been demonstrated that mutually exclusive changes in the expression of these antigens allow parasites to evade the host’s immune response. It is widely assumed that antigenic variation in protozoan parasites is accomplished by the spontaneous appearance within the population of cells expressing antigenic variants that escape antibody-mediated cytotoxicity. Here we show, both in vitro and in animal infections, that antibodies to Variant-specific Surface Proteins (VSPs) of the intestinal parasite *Giardia lamblia* are not cytotoxic, inducing instead VSP clustering into liquid-ordered phase membrane microdomains that trigger a massive release of microvesicles carrying the original VSP and switch in expression to different VSPs by a calcium-dependent mechanism. This novel mechanism of surface antigen clearance throughout its release into microvesicles coupled to the stochastic induction of new phenotypic variants not only changes current paradigms of antigenic switching but also provides a new framework for understanding the course of protozoan infections as a host/parasite adaptive process.

## Abstract (web summary)

Antigenic variation in protozoan parasites is considered a spontaneous process and antibodies to these variant antigens were cytotoxic. The clearance of the former antigen during antigenic switching is thought to occur by dilution of the original antigen. In this work, the authors provide in vitro and in vivo evidence, that low concentrations of antibodies against Variant-specific Surface Proteins (VSPs) of the intestinal parasite *Giardia lamblia* are not cytotoxic but induce a robust stimulation of the switching process and the release of the former antigen into extracellular microvesicles. This process is mediated by antibody-induced clustering of VSPs into L_o_-phase membrane microdomains through the raftophilic capability of the highly conserved transmembrane domain of the VSPs.

## Introduction

Antigenic variation (AV) is a mechanism developed by parasitic microorganisms to change the expression of their highly immunogenic surface molecules^1^. AV was discovered over a century ago^2^ and is considered an intrinsic process responsible for persistent and recurrent infections caused by unicellular microorganisms^1–3^.

*Giardia lamblia* colonises the lumen of the upper small intestine of vertebrates^4^. Its life cycle includes environmentally resistant cysts and proliferating trophozoites, which attach to the gut surface and cause the clinical manifestations of giardiasis^4^. Unlike other protozoan parasites, AV in *Giardia* was discovered as a phenomenon occurring in vitro^5^ (i.e., in the absence of any immune pressure) before being found to occur during animal infections^6^, suggesting that spontaneous antigenic switching is an inherent characteristic of this parasite^7^.

AV in *Giardia* involves VSPs, which cover the entire trophozoite surface and are the major antigens recognised by their hosts^4,7^. VSPs are integral membrane proteins with antigenically variable ectodomains containing multiple CXXC motifs and a highly conserved C-terminal region comprising a transmembrane domain (TMD) and a short cytoplasmic tail (CT)^8,9^. The parasite genome encodes a repertoire of 136 VSPs^9^, but only one is expressed on the surface of individual trophozoites at any time^8,9^. Expression of a unique VSP is regulated post-transcriptionally by an RNAi-like mechanism and maintained epigenetically^10^. However, why and how trophozoites change one VSP for another remains unknown.

As a major host response, the effect of antibodies against surface antigens has basically been studied in terms of their fate after binding to the cell surface. Many protozoa cope with surface-adhered antibodies by segregating them into “caps” or transferring the antigen/antibody complex to a “flagellar pocket” for endocytosis and lysosomal degradation, thereby avoiding protozoan agglutination and opsonization^11–13^. If antibody concentrations exceed the capability to remove them, parasites might die by either complement-dependent or independent antibody-mediated cytotoxicity^11–16^. Similarly, antibodies against *Giardia* VSPs have extensively been reported to be cytotoxic^5,7,15–19^.

AV occurs at very low rates in *Giardia* cultures^16^ and does not seem to occur in antibody-deficient hosts^20,21^. During infections in immunocompetent individuals, however, if antibody-mediated killing is as efficient as it was found in vitro, the number of switchers should be too low to sustain chronic infections. Therefore, we hypothesised that the relationship between protozoan parasites and the host immune response is more complex than previously speculated and that antibodies play a significant role during AV.

In this work, we found that low concentrations of antibodies against *Giardia*’s VSP stimulate antigenic switching and the release of the former antigen into extracellular microvesicles. These results show that antigenic variation is a host/parasite tunable process.

## Results

### Antibodies to VSPs stimulate switching

*Giardia* isolates are arranged into genetic groups or assemblages, each having a particular VSP repertoire^7–9^. When *Giardia* trophozoites are exposed to anti-VSP monoclonal antibodies (mAbs) or serum collected from infected animals, parasites agglutinate into large aggregates that have been considered clusters of dead cells^6–8,17–19^.

**Fig. 1:**
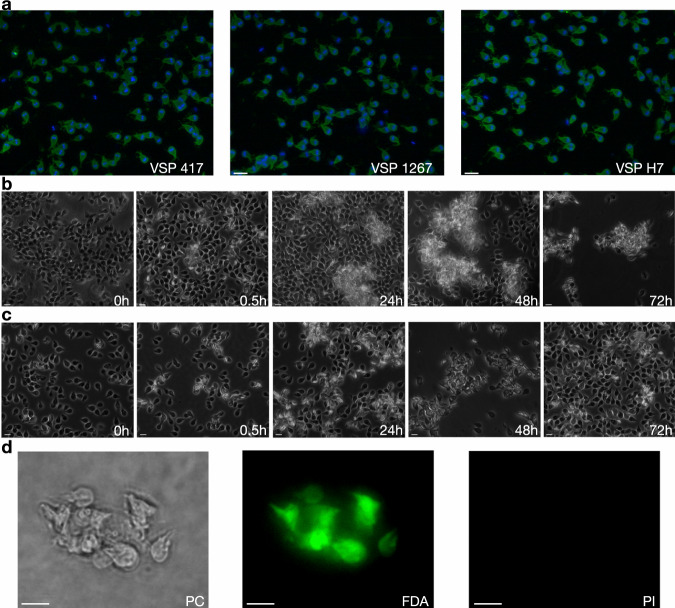
Effect of antibodies on *Giardia* trophozoites. **a** Representative immunofluorescence images of the different VSP clones (VSP, green; Nuclei, blue). **b** *Giardia* trophozoites of clone VSP417 incubated in the presence of 100 µM of mAb 7C2. A rapid agglutination of the cells was observed, and clusters of cells remained grouped but alive at 72 h of culture with mAb 7C2. **c** *Giardia* trophozoites of clone VSP417 incubated with 50 nM of mAb 7C2. A rapid agglutination of the cells was observed, but cells were then reattached to the glass tube at 72 h of culture with mAb 7C2. **d** Representative image of a cluster of *Giardia* trophozoites expressing VSP417, incubated for 48 h in the presence of mAb 7C2 (100 µM), and stained with FDA to label live cells (green) and with PI to label the nuclei of dead cells (red). All trophozoites of the aggregate are alive after that period in the presence of the anti-VSP antibody. Scale bars 10 µm.

**Fig. 2:**
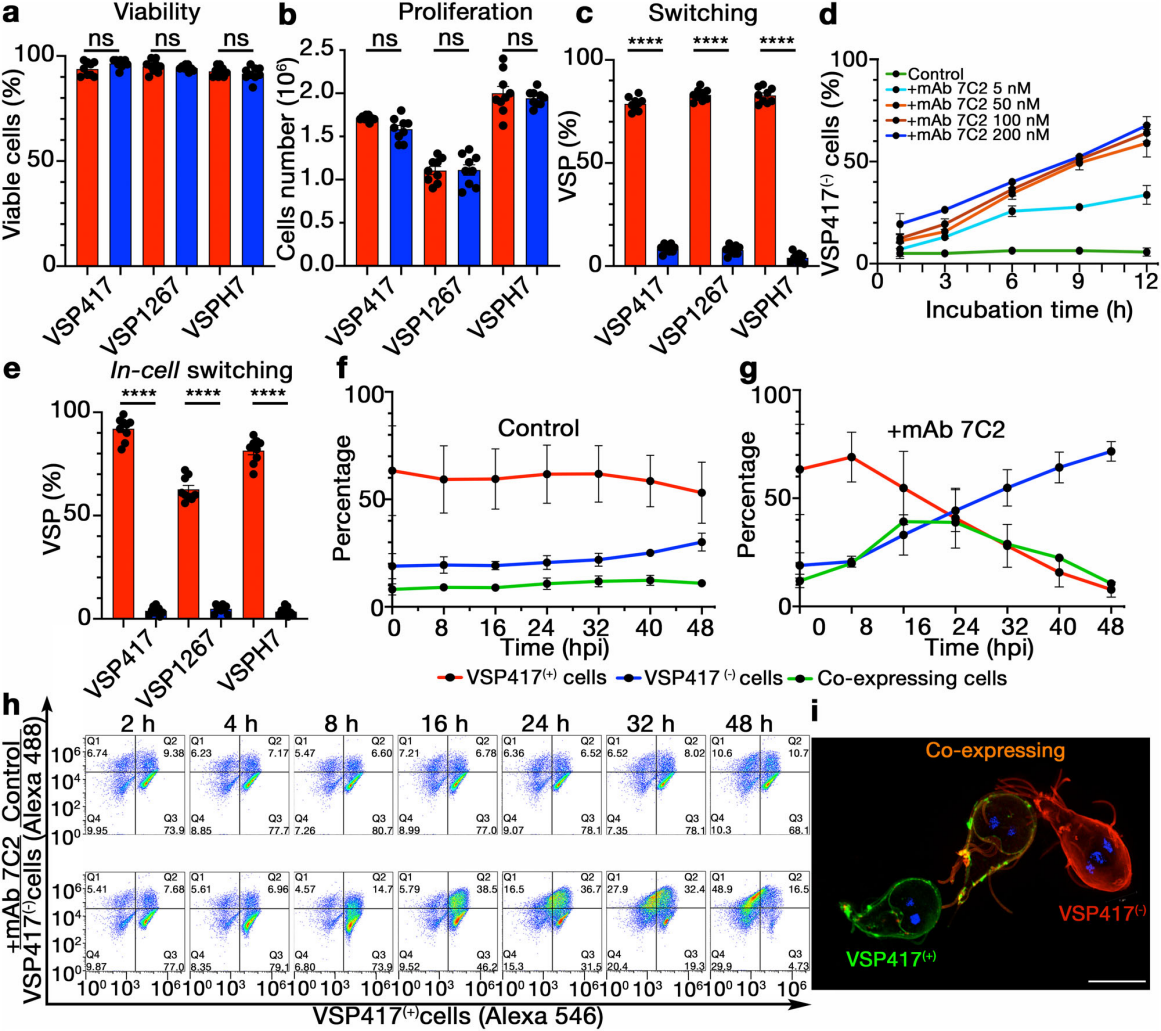
Antibodies to *Giardia* VSPs stimulate antigenic variation. Different VSP clonal populations were grown in the presence of 50 nM of either anti-CWP1 mAb (red columns, controls) or their cognate anti-VSP mAb (blue columns) for 72 h; then, the percentage of live cells (**a**), the total number of cells (**b**) and the percentage of the original VSP in each population (**c**) were determined. **d** Percentages of VSP417^(−)^ cells after discrete incubation times with mAb 7C2 at varying concentrations. **e** Percentage of cells expressing particular VSPs after *Giardia* cloning in the presence of their cognate anti-VSP mAb (blue) or the control (red). **f**, **g** Percentages of VSP417^(−)^, VSP417^(+)^ and co-expressing cells treated with mAb 7C2 (50 nM) for 48 h. **h** Representative cytograms showing the anti-clockwise distribution shift from VSP417^(+)^ to VSP417^(−)^ cells when cells were stimulated with mAb 7C2 (+mAb 7C2) at different hpi. Incubation with mAb 8F12 (Control) shows no redistribution. **i** Super-resolution structured illumination microscopy showing three trophozoites at different stages of antigenic switching after 24 h of mAb stimulation. Scale bar, 5 µm. Values represent mean ± s.e.m. of three independent experiments performed in triplicate. *p < 0.05; **p < 0.01; ***p < 0.001; ****p < 0.0001, ns not significant. Adjusted *p* values = **a** VSP417 = 0.1068, VSP1267 = 0.8990, VSPH7 = 0,7567; **b** VSP417 = 0.2081, VSP1267 = 0.9998, VSPH7 = 0.8007; **d** VSP417 = 0.1068, VSP1267 = 0.8990, VSPH7 = 0,7567. Statistical significance is based on one-way ANOVA on datasets with Sidak’s multiple comparisons tests.

To better explore the effect of antibodies to VSPs, specific mAbs against VSPs from trophozoites belonging to assemblages A1 (isolate WB/C6) and B (isolate GS/M-83) (Supplementary Table 1) were used to select by limiting dilution *Giardia* clones expressing either VSP417, VSP1267 or VSPH7. Once cloned (Fig. 1a), trophozoites were incubated with different concentrations of their specific anti-VSP antibodies. Results showed that at high concentrations (100 µM), purified mAbs produced rapid detachment of the parasites from the culture tubes and their clumping into aggregates containing live trophozoites, which remained grouped but motile for days (Fig. 1b and Supplementary movie 1). When the same clones were incubated for 72 h with lower concentrations of either their anti-VSP mAb or an unrelated mAb^22^ (50 nM), anti-VSP antibodies only produced a transient parasite detachment and agglutination (Fig. 1c), and neither their viability (Figs. 1d and 2a) nor proliferation (Fig. 2b) was affected. However, almost no trophozoite in the anti-VSP antibody-treated population maintained the expression of the original VSP, unlike the controls (Fig. 2c), indicating that low concentrations of anti-VSP antibodies may induce antigenic switching. Moreover, in clones treated with varying antibody concentrations and for different periods, the percentage of switchers incremented with both increasing induction times and antibody concentrations (Fig. 2d). Since experiments were performed in clones expressing different VSPs and using a variety of mAbs (Supplementary Table 1), the observed effects were not influenced by either the isotype of the anti-VSP mAb or their potential differences in affinity.

Then, two approaches were used to determine whether the observed AV was caused by induction of switching or antibody-mediated negative selection. First, trophozoites expressing a given VSP were re-cloned in a culture medium already containing either the mAbs directed to their specific VSP or the control antibody (50 nM). After 5 days, these *in-cell* experiments showed no differences in the number of growing clones (Supplementary Fig. 1). Since clones originated from a single trophozoite in contact with their anti-VSP mAb, the absence of antibody-induced negative selection was confirmed. In the controls, most clones maintained their VSPs. In contrast, in antibody-treated cells, the number of trophozoites expressing the original VSP was particularly low (Fig. 2e and Supplementary Fig. 1). Moreover, when the expression of different VSPs was examined by immunofluorescence assays (IFAs), all these newly expressed VSPs were present in small percentages, accounting for the absence of any order in the expression of the novel VSPs (Supplementary Table 2). Second, the differences in switching rates between clonal populations expressing either VSP417 or VSP1267 treated or not for 72 h with 50 nM of their corresponding anti-VSP mAbs were evaluated using an adapted Luria–Delbrück fluctuation test^23,24^. At these antibody concentrations and incubation times, results showed that anti-VSP antibodies induced a ~70-fold increase in the switching rate compared to the controls (Table 1). Besides, the fluctuation^23^ (variance/mean value) was >100 higher in untreated cells than in clonal parasites treated with its corresponding anti-VSP mAb, supporting that antibodies induce AV.

**Table 1:** Adapted Luria–Delbrück fluctuation test in *Giardia*

	C	Non-switchers	Switchers	N0	Nt	P0	m	Mean	Var.	Var./Mean	µ
**VSP417 + control**											
Exp. 1	12	949	51	10	5401	0,95	0,05	50,51	13107,46	259,52	9,60E−06
Exp. 2	12	949	51	10	5219	0,95	0,05	51,09	6944,47	135,93	1,00E−05
Exp. 3	12	945	55	10	5268	0,95	0,06	54,97	6000,55	109,16	1,07E−05
Exp. 4	12	939	61	10	5384	0,94	0,06	60,53	12259,69	202,52	1,16E−05
Exp. 5	12	935	65	10	5274	0,93	0,07	65,22	13824,60	211,96	1,28E−05
Exp. 6	12	946	54	10	5108	0,95	0,06	54,06	8954,31	165,63	1,09E−05
**VSP417 + mAb 7C2**											
Exp. 1	12	13	987	10	5683	0,01	4,34	986,93	48,25	0,05	7,63E−04
Exp. 2	12	13	987	10	5615	0,01	4,32	986,74	54,09	0,05	7,70E−04
Exp. 3	12	13	987	10	5142	0,01	4,37	987,30	48,46	0,05	8,49E−04
Exp. 4	12	13	987	10	5383	0,01	4,32	986,67	56,97	0,06	8,02E−04
Exp. 5	12	15	985	10	5093	0,02	4,17	984,52	86,19	0,09	8,18E−04
Exp. 6	12	18	982	10	5243	0,02	4,01	981,88	91,03	0,09	7,65E−04
**VSP1267 + control**											
Exp. 1	12	946	54	10	5422	0,95	0,06	53,73	11960,49	222,61	1,02E−05
Exp. 2	12	942	58	10	5491	0,94	0,06	57,71	11845,41	205,26	1,08E−05
Exp. 3	12	937	63	10	5526	0,94	0,07	63,15	12633,11	200,06	1,18E−05
Exp. 4	12	936	64	10	5453	0,94	0,07	64,27	11039,67	171,77	1,22E−05
Exp. 5	12	928	72	10	5586	0,93	0,08	72,46	12545,58	173,14	1,35E−05
Exp. 6	12	956	44	10	5404	0,96	0,05	44,49	8453,51	190,00	8,42E−06
**VSP1267 + mAb 7F5**											
Exp. 1	12	14	986	10	5668	0,01	4,28	986,13	50,81	0,05	7,55E−04
Exp. 2	12	13	987	10	5551	0,01	4,32	986,75	44,62	0,05	7,79E−04
Exp. 3	12	14	986	10	5450	0,01	4,29	986,33	53,96	0,05	7,88E−04
Exp. 4	12	13	987	10	5506	0,01	4,35	987,06	51,02	0,05	7,90E−04
Exp. 5	12	14	986	10	5563	0,01	4,30	986,47	52,51	0,05	7,73E−04
Exp. 6	12	14	986	10	5412	0,01	4,30	986,44	51,26	0,05	7,95E−04

Trophozoites of clones VSP417 and VSP1267 were cultured for 72 h in the absence (unrelated control mAb; Exp. 1–6) or the presence of either the anti-VSP417 mAb 7C2 (50 nM) or the antiVSP1267 mAb 7F5 (+mAb; Exp. 7–12) and subjected to the adapted Luria–Delbrück fluctuation test. In these experimental conditions, incubation with the mAb directed to the cognate VSP increased the switching rate (µ) by ~70 times compared to the controls treated with the unrelated mAb. C represents the total number of independent cultures, N0 is the number of parasites in the initial inoculum, Nt indicates the number of parasites at 72 h, P0 is the fraction of non-switchers, m is the –ln P0. Similar variance (Var.) and mean values indicate that the increase in µ is induced by the treatment.

To delve into the dynamics of VSP replacement, trophozoites expressing VSP417 were grown in the presence of mAb 7C2 or the unrelated mAb (50 nM). At different time points, the number of trophozoites labelled with mAb 7C2 and a polyclonal antibody against VSPs other than VSP417 (pAb VSP417^(−)^; Supplementary Fig. 2) was determined by IFAs and flow cytometry. IFAs results showed a decrease in the number of cells expressing the original VSP over time, with the concomitant increase of those simultaneously expressing the original and the novel VSPs and, finally, cells expressing only the new VSPs (Fig. 2f, g). Representative flow cytometry plots show that control cells exhibited no changes over a 72-h period; in contrast, mAb 7C2-treated trophozoites showed a conversion path from VSP417^(+)^ to VSP417^(−)^ cells throughout the treatment (Fig. 2h). This exchange was better observed in three different VSP417-expressing *Giardia* trophozoites incubated for 24 h with mAb 7C2, in which one is still expressing the original VSP, another has already switched to the expression of a new VSP, and the third parasite is still in the process of switching (Fig. 2i). This period of VSP colocalisation confirms the absence of negative selection; instead, there is an actual exchange of VSPs on the trophozoite membrane.

### Antibody-mediated VSP signalling

**Fig. 3:**
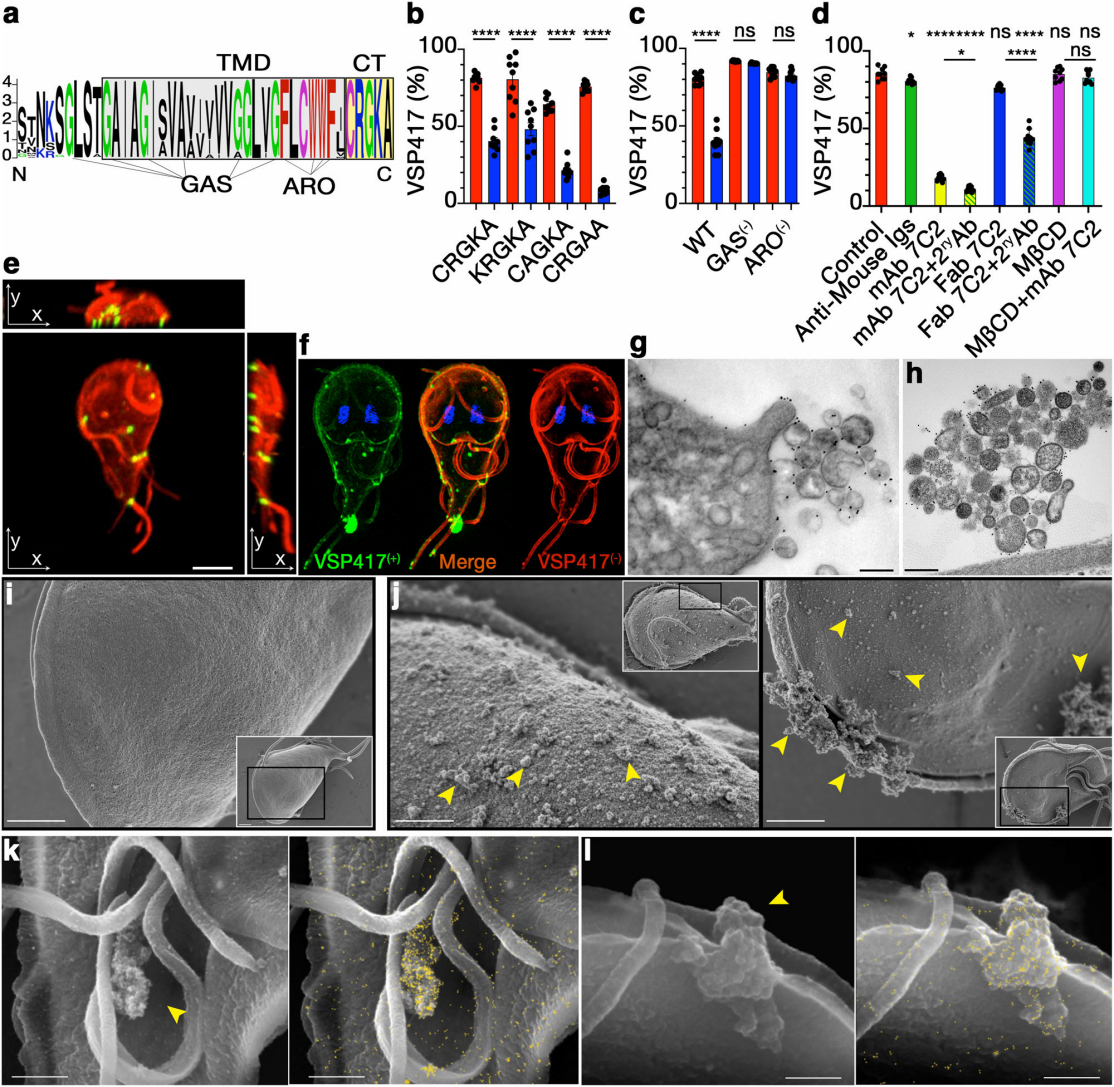
VSP signals through clustering by antibodies. **a** Sequence logo of the C-terminus of VSPs. TMD and CT are boxed in grey and yellow, respectively. G-xxx-G and small-xxx-small motifs (GAS) and aromatic residues (ARO) are indicated. VSP417 trophozoites coexpressing variants of the CT (**b**) and the TMD (**c**) of VSPH7 showing the percentage of VSP417^(+)^ cells after 72 h in the presence of either mAb G10/4 (blue) or the control (red). Values were normalised to the percentage of cells coexpressing both VSPs. **d** Percentage of VSP417-expressing cells after 72 h in the presence of mAb 7C2 (50 nM), Fab (100 nM) and Fab in combination with a secondary antibody (2^ry^ Ab), and in the presence or absence of MβCD (10 mM). **e** VSP417-expressing trophozoite after 1 h of treatment with mAb 7C2 (red); then fixed and incubated with mAb 7C2 (green). Maximum intensity projections on the three orthogonal axes from images taken in Z-stack show microdomains of VSP417 bound to the antibody (yellow) and free VSP417 (red). Scale bar 5 µm. **f** SR-SIM showing colocalisation (yellow) of the former VSP (VSP417, green) and the newly expressed VSP (VSP417^(−)^, red) after 24 hpi with mAb 7C2. TEM with gold labelling of a VSPH7 trophozoite incubated for 30 m with mAb G10/4 (**g**) and of a VSP417 trophozoite incubated for 30 min with mAb 7C2 (**h**). Scale bars, 500 nm. SEM images of a control cell (**i**) and mAb 7C2-treated trophozoites for 1 h (**j**). Insets are the cells from which a region (black box) was amplified. Arrowhead shows groups of microvesicles. **k**, **l** SEM of VSPH7 trophozoites incubated with gold-labelled mAb G10/4 for 30 min showing images obtained by secondary (left) and backscattered electrons (right). Arrows indicate groups of microvesicles containing VSPH7/mAb complexes (yellow dots). Scale bar 1 µm. Values represent mean ± s.e.m. of three independent experiments performed in triplicate. *p < 0.05; **p < 0.01; ***p < 0.001; ****p < 0.0001, ns not significant. Adjusted *p* values= **c** GAS = 0.6760 (ns), ARO = 0.3506 (ns); **d** Anti-Mouse Igs= 0.2224 (*); MBCD = > 0.9999 (ns); MBCD + mAb7C2 = 0.5042 (ns). Statistical significance is based on one-way ANOVA on datasets with Sidak’s multiple comparisons tests.

If antibodies against the different ectodomains of VSPs stimulated antigenic variation, VSPs might signal throughout their highly conserved C-terminal region, which comprises the TMD and a 5-amino acid CT (Fig. 3a).

Previous reports suggested the involvement of post-translational modifications of the CRGKA tail of VSPs, such as Cys-palmitoylation and Arg-citrullination in antibody-mediated cytotoxicity, and Lys-ubiquitination in VSP recycling^4, 17,18^. Therefore, the influence of the CT of VSPs during AV was evaluated in trophozoites simultaneously expressing two VSPs, which share their conserved C-terminal region but differ in their ectodomains. Trophozoites of the isolate WB endogenously expressing VSP417 were transfected to constitutively express VSPH7 of the isolate GS/M-83 (with or without mutations in the CT CRGKA) to function as “sensors” of the anti-VSP antibody, whereas VSP417 may serve as “reporter” of antigenic switching (Supplementary Fig. 3a). Trophozoites co-expressing VSP417 and VSPH7 with and without CT variants (Supplementary Fig. 3b) were then grown in the presence of mAb 7C2 to induce switching of VSP417, in the presence of mAb G10/4 to induce switching of VSP417 through binding to VSPH7, or with a mixture of both mAbs. When the CT of VSPH7 was either deleted (∆CT) or mutated to Alanines or Lysines, these variants were poorly expressed on the plasma membrane of the trophozoites (Supplementary Fig. 3c) and, therefore, these variants were not analysed further. After 72 h, mAb 7C2 (alone or in combination with mAb G10/4) promoted switching of VSP417 as expected (Fig. 3b and Supplementary Fig. 4). Still, mAb G10/4 also induced a significant switching of the reporter VSP417 in trophozoites co-expressing the VSPH7 wild type CT CRGKA as well as those having the CT variants KRGKA, CAGKA and CRGAA (Fig. 3b). These results suggest that, despite its conservation, the short CT of VSPs is not involved in signalling for AV.

Consequently, the highly homologous TMD of VSPs was investigated. A high degree of identity was found between the TMD of VSPs and those of surface antigens of other protists (e.g., *Trichomonas* spp., *Spironucleus* spp., and *Leishmania* spp.) and that of the mammalian carcinoembryonic antigen-related cell adhesion molecule 1 (CEACAM1)^25^ (Supplementary Fig. 3b). The similarity included length and presence of G-xxx-G and small(L/I/V)-xxx-small(L/I/V) motifs (GAS) and a domain rich in aromatic residues (ARO), all known to facilitate the formation of dimers and high-order oligomers^25–27^. Therefore, these motifs were disrupted by substituting the small amino acids of the TMD of VSPH7 for the larger amino acid Methionine and, separately, the aromatic residues were exchanged for Valines (Supplementary Fig. 3b). Then, the effects of mAb G10/4 in parasites co-expressing VSP417 and VSPH7 was analysed. TMD variants lacking either the GAS motifs or the ARO domain notably diminished VSP417 switching induced by the anti-VSPH7 antibody (Fig. 3c). Since these mutations changed the capability of TMD oligomerisation (Supplementary Fig. 5a), these results indicated that the TMDs of VSPs play an essential role in transducing the stimulus for AV.

In addition, since antibodies might facilitate VSP/VSP interactions, mAb 7C2 was fragmented to its corresponding Fabs and their relative ability to induce VSP417 switching was compared. Unlike the intact mAb (50 nM), Fab fragments (100 nM) failed to induce antigenic switching, but their efficiency was restored when Fabs were clustered with secondary antibodies (Fig. 3d), suggesting that VSP TMD oligomerisation and VSP clustering are crucial for switching.

TMD length and sequence are critical determinants of liquid-ordered phase (l_o_) membrane microdomains association^28^ and, indeed, CEACAM1 and oligomerised plasma membrane proteins are usually present on lipid raft-like structures^25–28^, as also are the GPI-anchored variable surface antigens of other protozoa^1,29^. In *Giardia*, the characteristics of VSP TMDs suggested that they would also translocate into l_o_-phase, lipid raft-like membrane domains previously described in this parasite^30,31^. In fact, when trophozoites were cultured in the presence of 10 mM of methyl-β-cyclodextrin (MβCD, a cholesterol-rich microdomain disrupting agent), VSP switching induced by anti-VSP mAbs was abolished (Fig. 3d). Hence, Triton X-100 lysates of trophozoites expressing VSP417 pre-treated or not with 50 nM of mAb 7C2 for 1 h were subjected to sucrose density gradient centrifugation to allow the isolation of low-density detergent-resistant membranes (DRM), which have properties of lipid rafts^32^. VSP417 was mainly detected in detergent soluble membranes (DSM) in treated and untreated trophozoites, but a fraction of the VSP417/mAb 7C2 complexes were redistributed into DRM upon antibody binding. Notably, pre-treatment of trophozoites with 10 mM of MβCD before DRM isolation avoided antibody-induced VSP417 redistribution into DRM (Supplementary Fig. 5b). These results are consistent with results of IFAs, which showed that at 1 h of incubation with the anti-VSP antibody, most of the VSP was still present all over the plasma membrane of the trophozoites, whereas the VSP/antibody complex localised into punctate domains (Fig. 3e). Therefore, the TMD of the VSPs may direct these proteins into cholesterol-rich microdomains upon antibody binding. Since l_o_-phase domains are dynamic structures, VSP-antibody interactions likely stabilise VSPs into larger platforms capable of recruiting cytoplasmic molecules that activate signal transduction mechanisms^30^.

### Antibody-bound VSPs are removed into microvesicles

To track the fate of the VSP bound to its specific antibody, *Giardia* clones expressing different VSPs were treated with their corresponding mAbs and analysed by structured illumination microscopy (SIM) 24 h post-induction (hpi). At the plasma membrane, the cognate VSP formed clusters and the newly expressed VSP showed a more homogeneous distribution (Fig. 3f). These microdomains were then studied at the ultrastructural level by combining transmission (TEM) and scanning (SEM) electron microscopy with or without immunogold-labelling. In Fig. 3g, h, immunogold TEM micrographs of a *Giardia* trophozoite at 30 mpi showed groups of microvesicles (MVs) on their surface. Although highly variable, the average size of these MVs was ~100 nm (Supplementary Fig. 6). By SEM, the cell surface changed from smooth in untreated trophozoites (Fig. 3i) to rough, full of MVs in antibody-treated cells (Fig. 3j). SEM with gold labelling showed that these MVs clumps were enriched in the target VSP (Fig. 3k, l). In contrast to the current assumption that the former surface antigen disappears by its continuous dilution in the plasma membrane after a new antigen is expressed, these results indicate that after antibody binding, VSPs are eliminated from the cell surface into MVs. In this process, called “Antigen Removal Coupled to Switching (ARCS)”, surface antigen clearance and antigenic variation are simultaneously triggered by surface antigen clustering into lipid raft-like microdomains.

**Fig. 4:**
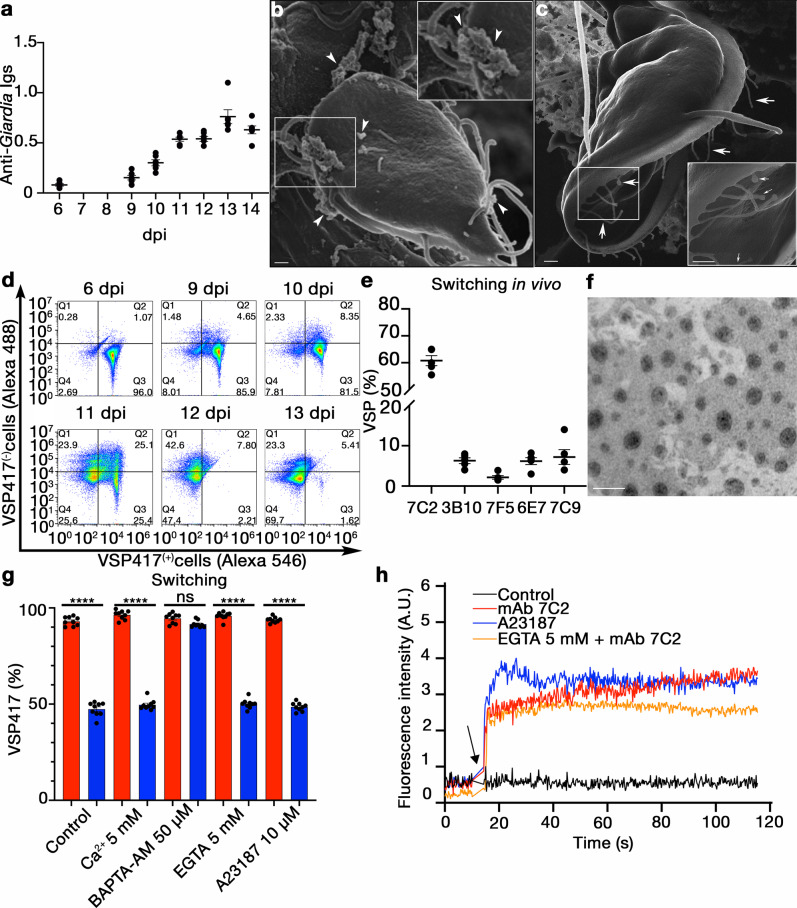
VSPs are released into microvesicles. **a** Total antibodies against *Giardia* VSP417- expressing trophozoites detected by ELISA in sera from infected gerbils at different dpi. Values represent relative optical density. **b**, **c** Extreme Resolution HI-SEM of VSP417 trophozoites recovered from the small intestine at 12 dpi. Extensive membrane projections emerging from the cell flange and the ventral disc are shown (arrows). A vesicle budding from a flagellum (arrowhead) and membrane projections at the end of a flagellum (asterisks) are observed. Scale bar 1 µm. **d** Representative cytograms showing the distribution shift from VSP417^(+)^ to VSP417^(−)^ populations during animal infections with a VSP417 clone. **e** Percentage of intestinal trophozoites recognised by different anti-VSP mAbs at 11 dpi. **f** Negative staining of purified microvesicles obtained trophozoites of clone VSP417 incubated with mAb 7C2 for 4 h. Scale bar, 200 nm. **g** Effects of different concentrations of extracellular calcium, EGTA, BAPTA-AM and the Ca^2+^ ionophore A23187 on antigenic switching of VSP417-expressing trophozoites grown in the presence of a control mAb (red) or of mAb 7C2 (blue) for 72 h. **h** The effects of anti-VSP mAb 7C2, a control antibody and the calcium ionophore A23187 on the intracellular levels of Ca^2+^ in VSP417-expressing cells are shown. Each line corresponds to the mean value of three independent experiments. Arrow indicates the time at which the stimulus was added. Values in other panels represent mean ± s.e.m. of three independent experiments performed in triplicate. *p < 0.05; **p < 0.01; ***p < 0.001; ****p < 0.0001, ns not significant. Adjusted *p* value= **g** BAPTA-AM 50 µM = 0.8151 (ns). Statistical significance is based on one-way ANOVA on datasets with Tukey’s multiple comparisons tests.

To test if a similar process occurs in vivo, gerbils (*Meriones unguiculatus*) were infected with VSP417-expressing trophozoites and intestinal parasites were collected and analysed by SEM and flow cytometry at different days post-infection (dpi). At the onset of the humoral immune response (Fig. 4a), identical protrusions and MVs seen upon antibody treatment in vitro were observed in trophozoites collected from the small intestine (Fig. 4b, c and Supplementary Fig. 7). Moreover, conversion from VSP417^(+)^ to VSP417^(−)^ cells was observed between 10 and 12 dpi, with a peak of original/new VSP co-labelling at 11 dpi (Fig. 4d). As observed in vitro, switching to different VSPs within the intestine was random (Fig. 4e). Notably, intestinal content of gerbils infected with trophozoites expressing VSP417 collected at 11 dpi (mainly containing sIgA) also induced switching of this clone in vitro (Supplementary Fig. 8).

To determine the composition of the MVs, proteomic analysis (Supplementary data 1) of purified MVs collected 4 hpi (Fig. 4f) demonstrated that their most enriched molecule is the original VSP. Except for multiple annexins (α-giardins^33^), PDC6IP^34^ (also known as ALIX) and components of the ESCRT machinery^35^, most proteins found in the antibody-induced MVs (Supplementary data 1) were similar to *Giardia* MVs artificially generated by incubation of the parasites with 2 mM of extracellular calcium^36^.

To test if the sole induction of MVs can trigger antigenic variation, clonal trophozoites were incubated with different extracellular [Ca^2+^] in either the absence or presence of anti-VSP antibodies. Neither viability nor proliferation was affected after 72 h of incubation, except in the presence of high concentrations of EGTA (>10 mM) (Supplementary Fig. 9a, b), but VSP switching was only induced when antibodies directed to their cognate VSP were present (Fig. 4g). Conversely, the intracellular Ca^2+^ chelator BAPTA-AM (50 µM) suppressed switching in trophozoites treated for 72 h with antibodies against their VSP (Fig. 4g and Supplementary Fig. 9c), without affecting parasite viability or proliferation (Supplementary Fig. 9a, b). To determine if VSP clustering by antibodies promotes an increase in the intracellular [Ca^2+^], trophozoites expressing VSP417 previously loaded with the calcium indicator Fluo 4-AM were subsequently incubated with either mAb 7C2 or a control mAb (50 nM). Results showed a substantial increase in the intracellular [Ca^2+^] soon after antibody binding to the VSP (Fig. 4h), in the presence or the absence of extracellular Ca^2+^. Remarkably, although the Ca^2+^ ionophore A23187 (10 µM) also increased the intracellular [Ca^2+^] to similar levels (Fig. 4h), it did not induce antigenic switching (Fig. 4g). This result suggests that an intracellular Ca^2+^ increase is necessary but insufficient to trigger ARCS and that the mechanical clustering of VSP caused by anti-VSP antibodies elicit a more complex process responsible for antigen elimination and switching. Since Ca^2+^ is essential for the function of annexins, PDCD6IP and PDCD6 (also known as ALG-2) during plasma membrane repair, virus budding and signal transduction^37–39^, these results suggest that anti-VSP antibodies promote the discharge of Ca^2+^ from intracellular stores to promote ARCS.

The fact that the TMD of VSPs makes these molecules raftophilic highlights the similarity to other protozoan variable surface antigens attached to the plasma membranes by GPI anchors^1,29,40^.

## Discussion

Antigenic variation seems to occur sporadically in protozoan cultures, which was interpreted as a spontaneous process that allows the switching of surface antigens after a given number of generations^1,41,42^. Unlike previously assumed^6,15–19^, antibodies against variant surface antigens are not cytotoxic at low concentrations; instead, they strongly stimulate antigenic switching.

Our findings show that variable surface antigens acquire the role of sensors of the humoral immune response, promoting antibody-directed phase separation of antigens into l_o_-phase membrane microdomains. The mechanical stress of the plasma membrane caused by antibody binding to raftophilic variable surface antigens then triggers the immediate clearance of the former antigen into extracellular MVs while stimulating antigenic switching. Interestingly, in the fish pathogenic ciliate *Ichthyophthirius multifiliis*, antibodies that cross-link its major variant surface antigens also induce a signalling pathway^43^. Authors reported that passive antibody transfer in vivo produces the rapid exit of some parasites from the infected host. Although it is unclear if antibodies induce antigenic variation, the GPI-anchored i-Antigens of *I. multifiliis* also work as a sensor of the humoral immune response, activating parasite motility and exit from the infected fish^43^.

Although the induction of microvesicles by different stress conditions has been observed in many protozoa^44–47^, including the formation of MVs in *Giardia* after exposure to anti-VSP antibodies^15^, their association with AV has never been investigated. Our novel observations that the original surface antigen is removed from the plasma membrane into microvesicles suggest that this process may divert the immune response to these MVs to allow the parasite to complete antigenic exchange before increasing concentrations of antibodies agglutinate the cells and/or exceed their endocytic capacity.

In contrast to negative selection, evidence for the induction of switching during antibody binding to surface antigens was observed in *Giardia* parasites, in which the simultaneous expression of the original and new surface antigen could be verified in the same cell during antigenic switching. In addition, the Luria–Delbrück fluctuation tests performed in *Giardia* show that the number of growing parasites is similar between parasites treated or not with the corresponding anti-surface antigen antibodies. However, in antibody-treated parasites the switching rate is strongly accelerated, indicating that antibodies induce switching instead of negative selection.

In the intestinal parasite *G. lamblia*, anti-VSP antibodies may cause not only antigenic switching at early stages of antibody production by the host but also parasite detachment from the gut epithelium when antibody concentrations increase during infections. Due to peristalsis, agglutinated trophozoites can thus reach the lower portions of the small intestine, where they differentiate into cysts^4^. Released cysts containing trophozoites already expressing different VSPs are prone to reinfect the same individual and expand the range of potential hosts^8,48^.

Since many pathogenic protozoa regulate the expression of a unique variant by post-transcriptional and epigenetic mechanisms^10,49,50^, antibodies might reset these control systems through an increase of intracellular [Ca^2+^]^51,52^, which may likely occur occasionally in culture. However, regardless of the mechanisms each pathogen uses to switch its surface antigens, one tempting hypothesis is that the antibody-stimulated release of MVs produces stochastic changes in the cytoplasmic levels of specific molecules in individual cells, resetting the epigenetically controlled antigen-expression program. This possibility might explain the stochastic nature of the switching response and the need for an increasing antibody exposure to complete antigenic switching of the whole parasite population^53^.

In sum, our results demonstrate a host-induced tuning of AV, which has significant consequences for understanding the course of protozoan infections and the propensity for multiple reinfections^54^.

## Methods

### Ethics

This research complies with all relevant ethical regulations. All procedures followed the protocols approved by the Institutional Committee for Care and Use of Experimental Animals (CICUAL protocols CIDIE.2016-36-15p-2, and CIDIE.2018-36-15p-3).

### *Giardia* cultures

*Giardia lamblia* assemblage A1 isolate WB (ATCC® 50803) clones and clone GS/M-83 (ATCC® 50581) (assemblage B) were cultured in TYI-S-33 medium, as previously described^55^. Clones expressing different VSPs were obtained by limiting dilution in 96-well culture plates placed in anaerobic chambers (AnaerogenTM Compact, Thermo Scientific® Oxoid®, Cat. # AN0010C) at 37 °C for 5 days and positive clones were then selected using specific anti-VSP mAb by immunofluorescence assay (IFA) as described^55^. Reactive clones were expanded in a culture medium overnight and tested for homogeneity before use^56^. For in-cell studies, these clones were re-cloned as described above, but in the presence of 50 nM of their specific anti-VSP mAb.

### Animals

BALB/c mice, Wistar rats and gerbils (6–8-week-old) of both sexes were housed in the vivarium of the CIDIE under specific pathogen-free (SPF) conditions in micro isolator cages (Techniplast^TM^), following NIH guidelines for laboratory animals.

### Infections with *G. lamblia*

Before infection, six-week-old gerbils were tested for the negativity of serum antibodies against *Giardia* antigens by ELISA, as previously reported^1^. Infections were carried out by orogastric administration of 2 × 10^5^ trophozoites expressing a particular VSP resuspended in 500 µl of PBS. Trophozoites from the intestine and the intestinal contents were collected on different days post-infection. Gerbils were sacrificed in a CO_2_ chamber and a 14-cm portion of the upper small intestine (measured from its junction to the stomach) was removed, dissected longitudinally using surgical scissors, incubated in 7 ml of PBS on ice for 30 min and vortexed to detach the parasites. The total number of parasites was counted from the intestinal content suspension using a Neubauer chamber. A centrifugation step at 1000 × *g* for 5 min was performed to isolate parasites from the intestinal content. PBS was discarded and replaced with culture medium at 37 °C. The parasites were incubated at 37 °C for 40 min to allow attachment to the walls of the culture tube; the medium was then removed along with the remains of intestinal content. Fresh medium was added, and the tubes were incubated on ice to detach the parasites for IFA and electron microscopy analyses.

### Production of monoclonal antibodies

Mouse mAbs to individual VSPs were generated as previously reported^57^. These antibodies’ isotype and light chain composition were determined using a commercial kit (mouse monoclonal antibody isotyping kit, dipstick format; Bio-Rad, Cat. # MMT1). Monoclonal antibody purification was carried out by FPLC using the ÄKTATM Pure 25 apparatus (GE Healthcare Life Sciences) coupled to a HiTrapTM Protein G HP (GE Healthcare®, Cat. # 29-0485-81), following the manufacturer’s protocol. After purification, a change from buffer to PBS was performed using the same equipment coupled to the HiPrepTM 26/10 desalting column (GE Healthcare, Cat. # 17-5087-01). Subsequently, protein concentration was measured using the BCA Protein Assay Kit (Pierce®, Cat. # 23225). The concentrations, expressed in molarity, are derived from the protein concentration present in the purified antibodies, considering a molecular weight of 150 kDa (for IgG). Purified antibodies were stored in small aliquots at −20 °C.

### Production of polyclonal antibodies

For rat anti-VSP417^(−)^ polyclonal antibody production, 8-week-old Wistar rats were immunised intraperitoneally on days 0, 14, and 28 with 50 µg of total protein extract of trophozoites derived from clones expressing VSP417 cultured during a week in the presence of 1 µM of mAb 7C2 to ensure complete switching to different VSPs. Before immunisation, protein extracts were emulsified in Sigma Adjuvant System (Sigma-Aldrich, Cat. # S6322). Rats were boosted intravenously on day 35 with 25 µg of the protein extract. Three days later, the rats were euthanised and total blood was withdrawn by cardiac puncture. Polyclonal serum was obtained and tested as non-reactive to VSP417-expressing trophozoites and reactive to most non-VSP417-expressing cells by IFA. VSP417^(−)^ pAb labelling for IFA and flow cytometry were performed on live, freshly collected trophozoites as described below.

### Flow cytometry

*Giardia* trophozoites were analysed by flow cytometry using a BD Accuri C6TM instrument (BD Biosciences®). Before acquiring the data, 8-point beads were used for manual quality control of the instrument. Unstained cells and compensation beads (BD Biosciences, CA) were used to set voltages and create single-stain negative and positive controls. Compensation was set to account for spectral overlap between the two fluorescent channels. No gating strategy was used but regions were set as quadrants reflecting positive staining for VSP417, positive staining for VSPs other than VSP417, and positive for both dyes. Density plots were displayed, indicating the percentage of each population. Data analysis and graph generation were performed using FlowJoTM 7.6 software (TreeStar®).

### Generation of Fab fragments

Fab fragments from mAbs 7C2 and 7F5 (both isotype IgG1) were generated using an antibody Fab preparation kit from Pierce® (Thermo Scientific®, Cat. # 44985) according to the manufacturer’s instructions. The Fab fragments were purified by FPLC using the ÄKTATM Pure 25 equipment coupled to a HiScree^TM^ MabSelec^TM^ SuRe^TM^ affinity column (GE Healthcare®, Cat. # 28-9269-77), according to the manufacturer’s protocol. The integrity of the binding sites of the Fab fragments was verified by IFA on *Giardia* trophozoites expressing the corresponding VSPs. SDS-PAGE confirmed the absence of undigested mAb under reducing and non-reducing conditions. The Fab concentrations expressed in molarity were derived from the protein concentration in the purified ones, considering a molecular weight of 50 kDa.

### Clonality assessment

For *Giardia*, IFA was performed as previously reported^55,56,58^. The percentage of cells expressing particular VSPs was calculated by counting 500 × 3 cells in triplicate experiments or by flow cytometry using an AccuriTM C6 flow cytometer (BD Biosciences®). For IFA, images were acquired using a Leica IRBE epifluorescence microscope with a 63X objective (using oil immersion; OA 1.4), equipped with an ORCA ER-II CCD camera (Hamamatsu). Images and videos were acquired using HCI Hamamatsu software. Image analysis and colocalisation were done with ImageJ software.

### Cytotoxicity and proliferation assays

*Giardia* trophozoites (1 × 10^4^) of clones VSP1267, VSP417 and VSPH7 were incubated with 50 nM of its corresponding mAbs (7F5, 7C2 and G10/4, respectively) for 72 h; then, 100 µl were used to analyse viability by propidium iodide/fluorescein diacetate staining^59^. The other 100 µl was used to count the total cell number in a haemocytometer.

### Populational switching assay

*Giardia* trophozoites (1 × 10^4^) of clones expressing VSP417, VSP1267 or VSPH7 were grown under the continuous presence of their cognate anti-VSP mAb (0.25–10 nM) in TYI-S-33 medium. After 1–3 days of culture, the number of trophozoites was quantified in a haemocytometer, and VSP characterisation was performed by IFA. Double Fab molar concentrations were used to ensure equal amounts of binding sites. For the transient-stimulus assays, 1 × 10^4^ trophozoites were treated with 50 nM of their corresponding anti-VSP mAb or mAb 8F12 or 100 nM of Fabs in TYI-S-33 medium for 1 h on ice, washed, and cultured under normal conditions for 3 days before VSP characterisation.

### *In-cell* switching assay

Fifty trophozoites expressing a particular VSP (VSP417, VSP1267, and VSP2B10) were diluted in 20 ml of TYI-S-33 medium containing 50 nM of the corresponding anti-VSP mAb and distributed in 96-well plates. The same procedure was followed in the TYI-S-33 medium with an unrelated mAb as a control (mAb 8F12). Plates were incubated for 5 days; then, the number of clones obtained was counted and IFA measured the percentage of the original VSP. For experiments with different incubation times and mAb concentrations, VSP417(+) trophozoites were treated with 0, 5–500 nM of mAb 7C2 in TYI-S-33 medium at 37 °C for 1, 3, 6 and 12 h. After incubation, VSP417 expression was determined by IFA.

### Switching rate determination in *Giardia*

An adapted Luria–Delbrück fluctuation test^24^ was designed to determine the *Giardia* switching rates in the presence or absence of the anti-VSP antibodies. Briefly, 10 trophozoites (N0) of *Giardia* clones expressing VSP417 were used to initiate 12 independent cultures of 1 ml each (C = 12) and cultured for an additional 72 h in the presence of anti-VSP417 mAb 7C2 (*n* = 6) or of the unrelated mAb 8F12 (*n* = 6) at 50 nM. Subsequently, cells from each well were counted (Nt), washed and resuspended in 2.4 ml of culture medium without mAbs and plated into 12 new wells. After 24 h, trophozoites were collected from all wells and subjected to IFAs using Alexa Fluor 488-conjugated anti-VSP mAbs 7C2 and 1000 parasites per well were counted to record positive (non-switchers) and negative (switchers) cells. Then, the P0 method^23,24^ was used to calculate the VSP switching rates (µ), where P0 is the fraction of non-switchers per culture. The number of switchers per culture (m) was considered the -ln P0. The switching rate was calculated as m divided by the number of cells per culture at 72 h of incubation with the antibodies (Nt). The mean and the variance in the number of switchers were also determined. When the variance and the mean are similar, their ratio represents inducible cultures, whereas higher values correspond to spontaneous switching^23,24^.

### In vitro switching dynamics

*G. lamblia* trophozoites of clone VSP417 were grown in TYI-S-33 medium with 50 nM of mAb 7C2 or the unrelated mAb in 8-ml culture tubes. The initial number of trophozoites used for each chosen time point was calculated to reach a final number of 3 × 10^6^ cells after the stipulated culture time, assuming a generation time of 8 h, to ensure exponential growth. At each time point, trophozoites were counted and treated with Alexa Fluor^TM^−488 direct-labelled 7C2 mAb (Alexa Fluor^TM^−488 labelling kit; Invitrogen^TM^, Cat. # A10235) and VSP417^(−)^-pAb in TYI-S-33 medium on ice for 40 min. The cells were washed twice with ice-cold phosphate-buffered saline (PBS) and fixed with 4% formaldehyde (PFA) in sodium phosphate 0.1 M pH 7.2 for 1 h. Then, cells were washed twice with PBS and treated with ammonium chloride 50 mM for 10 min and with 1% BSA in PBS (blocking solution) for 15 min. Labelling for cell cytometry was performed using goat anti-mouse IgG (H-L)-PE (Invitrogen^TM^, Cat. # PA1-84395, 1/6000), goat anti-rat IgG (H-L)-biotin 1/2000 (Invitrogen^TM^, Cat. # A18869), and streptavidin-Alexa Fluor^TM^ 488 (Invitrogen^TM^, Cat. # S11223, 1/6000) in blocking solution. Super-resolution structured illumination microscopy (SR-SIM) analysis was performed on trophozoites at 24 h post-induction. Fixed trophozoites were attached to a coverslip using poly-L-lysine solution 0.1% (Sigma-Aldrich, Cat. # P8920), blocked and treated with goat anti-mouse IgG (H + L)-Alexa Fluor^TM^ 488 (Invitrogen^TM^, Cat. # A11001) and goat anti-rat IgG (H-L)-Alexa Fluor^TM^ 546 (Invitrogen^TM^, Cat. # A11081) secondary antibodies at a dilution of 1/200 in blocking solution for 1 h. DNA staining was performed with DAPI. Images were taken using a Zeiss Elyra PS1 microscope system. Images were acquired with five grid rotations and analysed with the ZEN software (Zeiss).

### In vivo switching dynamics

Gerbils were infected by orogastric inoculation of 2 × 10^5^ trophozoites of clone VSP417 resuspended in 0.5 ml of PBS. Randomly selected gerbils were euthanised on days 6, 9, 10, 11, 12, 13 and 14 post-infection. The first 14 cm portion of the small intestine was isolated and dissected longitudinally before being incubated on ice-cold PBS for 30 min. The supernatants were collected, *Giardia* trophozoites were quantified in a haemocytometer, and VSP characterisation was carried out by flow cytometry, as explained above.

### Cloning and transfection

The plasmid pTUBpac8 was used to express cytoplasmic tail-variants of VSPH7. To construct the different VSPH7 variants, the same forward primer (fwd, 5′-CAT GCC ATG GAT GTT TCT ATT AAT TAA TTG CCT A-3′) was used in combination with different reverse primers. VSPH7WT (wt_rev, 5′-GCG GAT ATC CGC CTT CCC GCG GCA GAC GAA-3′) is the wild-type version of the VSPH7 (CRGKA); VSPH7AxK (ak_rev, 5′-GCG GAT ATC CGC CGC CCC GCG GCA GAC GAA-3′) has Ala instead of a Lys in the tail. Similar modifications were made in the variants VSPH7AxR (ar_rev, 5′-GCG GAT ATC CGC CTT CCC CGC GCA GAC GAA-3′) and VSPH7KxC (kc_rev, 5′-GCG GAT ATC CGC CTT CCC GCG CTT GAC GAA-3′), where Arg was replaced with Ala and Cys was replaced with Lys, respectively. For TMD variants, the pTUBH7HApac was modified for the repositioning of the ApaI sequence from the 5´end of the VSPH7 to a new position upstream of the TMD sequence. PCR mutagenesis by overlap extension was performed using the following primers: Fwd_A, 5′-GAA CCA TGG GGC TCT TAA TTA ATT G-3′; Rev_B, 5′-GAG GAG AGG TTG GGC CCA CTA-3′; Fwd_C, 5′-TAG TGG GCC CAA CCT CTC CTC-3′; and Rev_D, 5′-AAT TCA CTG CGG CCG CAA CTC-3′. The vector obtained was named pTUBH7ApaI. Then, two VSPH7 TMDs variants were obtained through the annealing of the following primers: Fwd_GAS/M, 5′- C AAC CTC TCC TCT ATG GCG ATC GCA ATG ATC TCG GTG ATG GTC ATT GTC GTC GTC ATG GGC CTC GTC ATG TTC CTC TGC TGG TGG TTC GTC TGC CGC GGG AAG GCG TGA GC −3′; Rev_GAS/M, 5′- GGC CGC TCA CGC CTT CCC GCG GCA GAC GAA CCA CCA GCA GAG GAA AAG GAC GAG GCC AAG GAC GAC GAC AAT GAC GGC CAC CGA GAT AAG TGC GAT CGC AAG AGA GGA GAG GTT GGG CC-3′; Fwd_Aro/V, 5′-CAA CCT CTC CTC TGG CGC GAT CGC AGG CAT CTC GGT GGC CGT CAT TGT CGT CGT CGG AGG CCT CGT CGG CGT TCT CTG CGT TGT TGT TGT CTG CCG CGG GAA GGC GTG AGC-3′; and Rev_Aro/V, 5′-GGC CGC TCA CGC CTT CCC GCG GCA GAC AAC AAC AAC GCA GAG AAC GCC GAC GAG GCC TCC GAC GAC GAC AAT GAC GGC CAC CGA GAT GCC TGC GAT CGC GCC AGA GGA GAG GTT GGG CC-3′. In the GAS/L TMD, Gly residues were replaced with Met, and in the ARO TMD, Trp and Phe residues were replaced with Val. To introduce TMD variants into the pTUBH7ApaI vector, both the vector and TMDs were digested using ApaI and NotI and ligated. Deletion and mutations were confirmed using dye terminator cycle sequencing (Beckman Coulter). Trophozoites were transfected by electroporation and selected with puromycin (InvivoGen, Cat. # Ant-pr-5), as previously described^60^.

### In silico VSP TMD oligomerisation models

The oligomerisation capability of the TMD was analysed with THOIPA^61^ and PREDIMMER^62^ and visualised with VMD^63^. Alignments were performed with Clustal Omega^64^ and WebLogo^65^. Figures and graphic representations were created with BioRender.com.

### Isolation of detergent-resistant membranes (DRMs)

DRMs were prepared as previously described^66^, with modifications. Trophozoites (2.5 × 10^7^) of clone VSP417 were resuspended in 5 ml of TYI-S-33 medium with 50 nM of mAb 7C2 or without mAb at 37 °C for 30 min. Pre-treatment with 10 mM methyl-β-cyclodextrin (MβCD; Sigma-Aldrich Cat. # 128446-36-6) was performed in PBS supplemented with ascorbic acid (0.01% w/v) and cysteine (2% w/v), pH 7.2 (PBSAC) for 30 min and then incubated with 50 nM of mAb 7C2 in PBSAC. Trophozoites were then washed with TNE buffer (50 mM Tris-HCl pH 7.5; 150 mM NaCl; 1 mM EDTA) and lysed in 500 µl of TNE buffer supplemented with 1% w/v Triton X-100 and 2X cOmplete™ protease inhibitor cocktail (Merck®, Cat. # 11836145001) on ice for 30 min. An equal volume of ice-cold TNE 85% sucrose was added to cell lysates to adjust sucrose concentration at 42.5%. The lysates were then placed at the bottom of a 13.2-ml ultra-clear centrifuge tube (Beckman) and carefully overlaid with 5 ml of 35% sucrose in TNE and 1 ml of 5% sucrose in TNE. Tubes were centrifuged at 250,000 × *g* in an SW41Ti rotor (Beckman Coulter) at 2 °C for 20 h. Fourteen fractions of 500 µl were carefully obtained from the top of each tube and 36 µl of each fraction was subjected to SDS-PAGE under non-reducing conditions. VSP417 was detected by Western blotting using mAb 7C2 (1:1,000) and peroxidase-conjugated goat anti-mouse IgG (H-L) (Invitrogen^TM^, Cat. # 626520; 1:5000). Fractions 3–12 and 13–14 were considered DRM and DSM, respectively.

### Dynamics of antibody-bound VSPs elimination

For electron microscopy, 5 × 10^6^ trophozoites of clones VSP417, VSP1267 and VSPH7 were treated with 2.5 nM of their cognate anti-VSP mAbs in TYI-S-33 medium on ice for 1 h. Then, cells were washed three times with ice-cold PBS, resuspended in 7 ml of TYI-S-33 medium and incubated at 37 °C for 0, 15, 30 and 60 min. At each time point, trophozoites were placed on ice for detachment, washed twice with ice-cold filtered PBS, and fixed with 4% FA in sodium phosphate 0.1 M pH 7.2 for 1 h. Cells were washed twice with PBS and treated with ammonium chloride 50 mM for 10 min and with blocking solution for 15 min. Immunogold labelling was performed by treating the trophozoites with goat anti-mouse IgG-(H-L)-gold 10 nm (Abcam, Cat. # ab39619) in a dilution of 1/10 in blocking solution for 2 h and fixed again with 2.5% glutaraldehyde in 0.1 M cacodylate buffer (pH 7.2).

### Scanning electron microscopy (SEM) and Helium ion microscopy (HM)

Briefly, glutaraldehyde-fixed cells were post-fixed in 1% OsO4 for 15 min. Then, samples were dehydrated in crescent series of ethanol up to 100%, critical point-dried with liquid CO2 and sputter-coated with carbon to observe the cell surface in detail. The specimens were examined in a Quanta^TM^ SEM (FEI Co., The Netherlands) equipped with FEG filament. Images were obtained via secondary electron (SE) and/or backscattered electron (BSE) detection at an accelerating voltage of 15 kV. For High-Resolution Scanning Microscopy analysis, the sputter-coating step was performed using a thin layer (2 nm) of platinum, and cells were observed on an Auriga^TM^ High-Resolution SEM (Zeiss). For HM, cells were processed as described above and observed, without any subsequent coating, under a Zeiss Orion Helium Ion Microscope.

### Transmission electron microscopy (TEM)

Glutaraldehyde-fixed cells were post-fixed with 1% OsO4 and 0.8% potassium ferrocyanide for 40 min. The samples were dehydrated in crescent grades of acetone up to 100% and embedded in epoxy resin. Ultrathin sections (50–60 nm thick) were cut, collected and stained with uranyl acetate and lead citrate. Lastly, the samples were analysed using a Tecnai^TM^ Spirit TEM (FEI Co.).

### Microvesicle purification and validation

Exponentially growing trophozoites (1.5 × 10^8^) of clone VSP417 were washed twice with filtered PBS at 37 °C and incubated 4 h at 37 °C in the absence or the presence of mAb 7C2 (50 nM) in microvesicle purification medium (ultrafiltrated TYI-S-33 medium, 100 kDa MWCO, supplemented with 3% adult bovine serum previously ultracentrifuged overnight at 250,000 × *g*). Tubes were ice-cooled and then centrifuged in an SW41Ti rotor (Beckman) at 4 °C for 10 min. Supernatants were collected in 50-ml centrifuge tubes and centrifuged at 3000 × *g* at 4 °C for 40 min; this step was repeated once. Then, the supernatants were concentrated 10X in a centrifugal filter device (Centricon^TM^ Plus-70-100K, Millipore®, Cat. # UFC710008) and ultracentrifuged in a Beckman® SW41Ti rotor (25,000 rpm, 2 h, at 4 °C). Pellets were washed in an equal amount of filtered ice-cold PBS and ultracentrifuged again to recover the MVs. In the absence of mAb, no MVs were detected and, consequently, untreated samples were discarded for subsequent analysis. The size of the microvesicles was determined by NanoSight analysis as in Evan-Osses et al.^36^. Purification of MVs was validated by TEM. Briefly, a pellet of microvesicles was resuspended in 500 µl of 1% BSA in PBS with anti-mouse IgG-Gold 10 nm (Abcam, Cat. # ab39619; 1:100) and incubated on ice for 2 h. Next, the samples were washed twice with filtered PBS and fixed with 2% PFA in PBS. Formvar-coated nickel grids were floated on 10 µl of microvesicle suspension for 20 min to obtain TEM images for 20 min. Subsequently, microvesicles adsorbed on the grids were post-fixed in 1% glutaraldehyde. The grids were rinsed with dH2O and contrasted successively in 2% uranyl acetate pH 7 and 2% methylcellulose/0.4% uranyl acetate, pH 4. Microvesicles were visualised using a Leo 906-E (Zeiss) transmission electron microscope.

### Proteomics of purified microvesicles

Monoclonal antibody 7C2-induced microvesicles from three independent *Giardia* clones expressing VSP417 and treated with the mAb 7C2 were analysed with MS/MS. Purified microvesicles were lysed in RIPA buffer with cOmplete™ protease inhibitor cocktail (Merck®, Cat. # 11836145001). Fifteen µg of each sample were run 1 cm in SDS-PAGE under non-reducing conditions. The following procedure and analysis were done by MSBioworks.com. In-gel digestion was then performed on each sample using a robot (ProGest, DigiLab) with the following protocol: washed with 25 mM ammonium bicarbonate followed by acetonitrile, reduced with 10 mM dithiothreitol at 60 °C followed by alkylation with 50 mM iodoacetamide, digested with sequencing grade trypsin (Promega) at 37 °C for 4 h and quenched with formic acid. Half of each digested sample was analysed by nano LC-MS/MS with a Waters NanoAcquity HPLC system interfaced to a Thermo Fisher Q Exactive mass spectrometer. Peptides were loaded on a trapping column and eluted over a 75-μm analytical column at 350 nL/min; both columns were packed with Luna C18 resin (Phenomenex). The total instrument time used was 2 h. The mass spectrometer was operated in data-dependent mode, with the Orbitrap operating at 70,000 FWHM and 17,500 FWHM for MS and MS/MS, respectively. The 15 most abundant ions were selected for MS/MS. Data were processed using Mascot (Matrix Science) with the following parameters: Enzyme, Trypsin/P; Database, NCBI Giardia lamblia RefSeq 2.1 proteome (concatenated forward and reverse plus common contaminants); Fixed modification, Carbamidomethyl (C); Variable modifications, Oxidation (M), Acetyl (N-term), Pyro-Glu (N-term Q), Deamidation (N/Q); Mass values, Monoisotopic; Peptide Mass Tolerance, 10 ppm; Fragment Mass Tolerance, 0.02 Da; Max Missed Cleavages, 2. Mascot DAT files were parsed into Scaffold (Proteome Software) for validation, filtering and creating a non-redundant list per sample. Data were filtered using 1% protein and peptide FD, requiring at least two unique peptides per protein. The mass spectrometry proteomics data have been deposited to the ProteomeXchange Consortium via the PRIDE partner repository^67^ with the dataset identifier PXD031141 and 10.6019/PXD031141.

### Calcium treatments and quantification

The involvement of calcium was determined by incubating 1-2 × 10^4^ trophozoites of clone VSP417 in PBS and stimulated in a culture medium with different concentrations of CaCl2 (0.1–10 mM) at 37 °C for 72 h in the presence or absence of their cognate anti-VSP mAb; then, the percentage of the former VSP was determined in the parasite population by IFA. On the other hand, the effect of the intracellular calcium chelator during AV induced by antibodies was studied. Giardia trophozoites (1 × 10^6^) of clone VSP417 were incubated in culture medium supplemented with 1, 25 or 50 µM of BAPTA-AM (Sigma-Aldrich, Cat. # A1076) at 37 °C for 72 h, in the presence of 50 nM of mAb 7C2 or an unrelated antibody. After that period, parasites were washed twice in cold PBS, collected by centrifugation, and analysed using IFA. For intracellular calcium measurements, Fluo 4-AM was freshly prepared in dehydrated DMSO before each experiment. Trophozoites expressing VSP417 were harvested by centrifugation (1000 × *g* for 5 min) and the cells were then resuspended in Hanks’s balanced salt solution (HBSS) supplemented with 10 mM HEPES. Cells were then incubated with Fluo 4-AM (10 µM) and Pluronic F-127 (0.04%) at room temperature for 30 min and then washed three times for at least 15 min to allow complete intracellular de-esterification of the dye. Finally, trophozoites were incubated for 30 min at 37 °C in HBSS-HEPES. Kinetic experiments were performed with a Cary Eclipse spectrofluorimeter (Agilent Technologies®) equipped with a stirred cuvette holder. A 0.3 mm path cuvette was used. Trophozoites were excited at 495 nm and emission at 520 nm with excitation/emission slit widths of 5/5 nm.

### Statistical and reproducibility

No statistical methods were used to predetermine sample size, except for the Luria–Delbrück fluctuation tests. The experiments were randomised. The investigators were blinded to allocation during experiments and outcome assessment. Average (mean) and s.e.m. were calculated in Excel. Statistical significance is based on one-way or two-way ANOVA on datasets with Tukey’s and Sidak’s multiple comparisons tests or Bonferroni post-test, respectively, using GraphPad (Prism). Results in Figs. 1a, d, 2i, 3e–l; 4b, c, f, Supplementary Figs. 2, 3c, 5b, 7; 8a, b are representative results of three independent experiments. All figures show the mean value ± s.e.m of three independent experiments performed in triplicate. Statistically differences are indicated in each graph as *p < 0.05, **p < 0.01, ***p < 0.01, ****p < 0.001 and ns not significant.

### Reporting summary

Further information on research design is available in the Nature Portfolio Reporting Summary linked to this article.

## Supplementary information

Suplementary Information

Peer Review File

Description of Additional Supplementary Information

Supplementary movie 1| Anti-VSP antibodies induce rapid detachment and agglutination of trophozoites without killing the parasites.

Supplementary data 1| Proteomic analysis of purified microvesicles.

Reporting Summary

## Data Availability

Source data are provided in this paper. All other data are available in the main text or the supplementary materials. Proprietary antibodies are available upon request through a material transfer agreement (MTA). The mass spectrometry proteomics data have been deposited at the ProteomeXchange Consortium via the PRIDE partner repository with the dataset identifiers PXD031141. Source data are provided with this paper.

## Source data

Source Data
