# Supplementary material for: Antibodies to variable surface antigens induce antigenic variation in the intestinal parasite Giardia lamblia
Source: Nat Commun. 2023 May 3;14:2537. doi: 10.1038/s41467-023-38317-8 (PMC10156722; doi:10.1038/s41467-023-38317-8)
Supplement: Supplementary file 1 — Suplementary Information [file 41467_2023_38317_MOESM1_ESM.pdf]

Supplementary information

Antibodies to variable surface antigens induce antigenic variation in the intestinal parasite *Giardia lamblia*

Supplementary Tables and Figures

Supplementary Table 1| Murine monoclonal antibodies against different *Giardia* VSPs and CWP1. Monoclonal antibodies (mAbs) specific for a given VSP of assemblages A1 (WB isolate) or B (GS/M-83 isolate). For VSP1267, two different mAbs were tested (IgG_1 and IgM). The mAb against Cyst Wall Protein 1 (CWP1; a *Giardia* protein not expressed in proliferating trophozoites) was used as a control.

mAb	Isotype	Protein/Locus Tag/GenBank ID	Assemblage	Isolate
**5C1**	IgM	VSP1267/GL50803_00112208/XP_001706567.1	A1	WB
**7F5**	IgG_1	VSP1267/GL50803_00112208/XP_001706567.1	A1	WB
**7C2**	IgG_1	VSP417/GL50803_00113797/XP_001710078.1	A1	WB
**9B10**	IgG_1	VSP9B10/GL50803_00101074/XP_001706983.1	A1	WB
**2B10**	IgM	VSPAS8/GL50803_0040591/XP_001708393.2	A1	WB
**7C9**	IgM	VSP7C9/GL50803_0050375/XP_037901545.1	A1	WB
**6E7**	IgG_1	VSPA6/GL50803_00221693/XP_001707734.2	A1	WB
**G10/4**	IgG_1	VSPH7/GSB_150963/ESU41081.1	B	GS
**8F12**	IgG_{2a}	CWP1/GL50803_005638/XP_001704890.1	A1	WB

Supplementary Table 2| Expression of novel VSPs after mAb-induced antigenic switching. Two 96-well plates were used to distribute 0.5 cells per well of clone VSP417. After 5 days of culture in the presence of mAb 7C2 (50 nM), the composition of *Giardia* populations for 20 randomly selected wells was determined by IFA using mAbs against different VSPs. Values are percentages of positivity to the corresponding anti-VSP mAb.

**Plate A**	**mAb**					
**Well**	**7C2**	**9B10**	**7F5**	**6E7**	**7C9**	**2B10**
**1**	0.0	9.5	1.7	1.1	2.6	1.1
**2**	0.0	7.9	1.6	1.2	0.7	1.3
**3**	0.0	6.2	2.2	1.4	5.3	9.1
**4**	0.0	1.3	0.9	14.1	2.3	2.2
**5**	0.0	4.6	2.1	0.9	14.2	2.9
**6**	0.0	3.2	0.7	0.8	9.2	6.3
**7**	0.0	1.6	3.3	3.8	4.1	5.3
**8**	0.0	4.4	14.1	0.5	2.7	0.2
**9**	0.0	5.5	1.6	6.2	9.3	6.2
**10**	0.0	2.1	0.7	1.4	2.1	3.2
**11**	0.0	4.3	1.8	2.1	2.5	2.8
**12**	0.0	18.1	1.3	4.9	1.8	1.5
**13**	0.0	0.9	0.6	4.7	1.5	2.8
**14**	0.0	6.3	4.2	1.8	5.4	5.4
**15**	0.0	1.2	1.2	0.1	2.1	6.3
**16**	0.0	9.3	8.6	0.2	4.1	1.3
**17**	0.0	1.2	2.3	0.9	0.0	3.2
**18**	0.0	2.6	2.1	0.1	2.3	2.5
**19**	0.0	12.2	0.2	0.2	2.1	1.0
**20**	0.0	0.3	7.0	2.3	9.9	2.5

**Plate B**	**mAb**					
**Well**	**7C2**	**9B10**	**7F5**	**6E7**	**7C9**	**2B10**
**1**	0.0	2.4	1.3	1.7	8.0	4.6
**2**	0.0	6.3	9.7	3.0	8.3	1.5
**3**	0.0	0.0	1.1	7.2	5.8	1.3
**4**	0.0	1.3	0.9	0.7	1.5	3.0
**5**	0.0	1.4	3.1	2.9	2.1	2.0
**6**	0.0	6.3	2.1	3.8	0.7	7.6
**7**	0.0	4.2	3.6	6.0	9.2	1.4
**8**	0.0	2.1	6.7	6.8	9.1	0.4
**9**	0.0	1.1	3.4	5.0	0.3	1.1
**10**	0.0	3.3	1.6	3.6	0.5	0.4
**11**	0.0	0.2	0.5	4.7	1.6	0.9
**12**	0.0	1.2	0.2	1.3	4.5	0.8
**13**	0.0	4.3	9.7	4.1	5.4	6.1
**14**	0.0	0.0	2.2	2.1	8.9	4.1
**15**	0.0	5.2	1.0	6.9	4.8	2.5
**16**	0.0	7.0	7.1	0.6	6.2	4.0
**17**	0.0	4.1	1.8	6.2	3.4	0.5
**18**	0.0	11	0.0	8.1	4.4	3.1
**19**	0.0	7.2	0.0	0.5	4.8	1.5
**20**	0.0	3.4	0.0	3.3	3.2	8.7

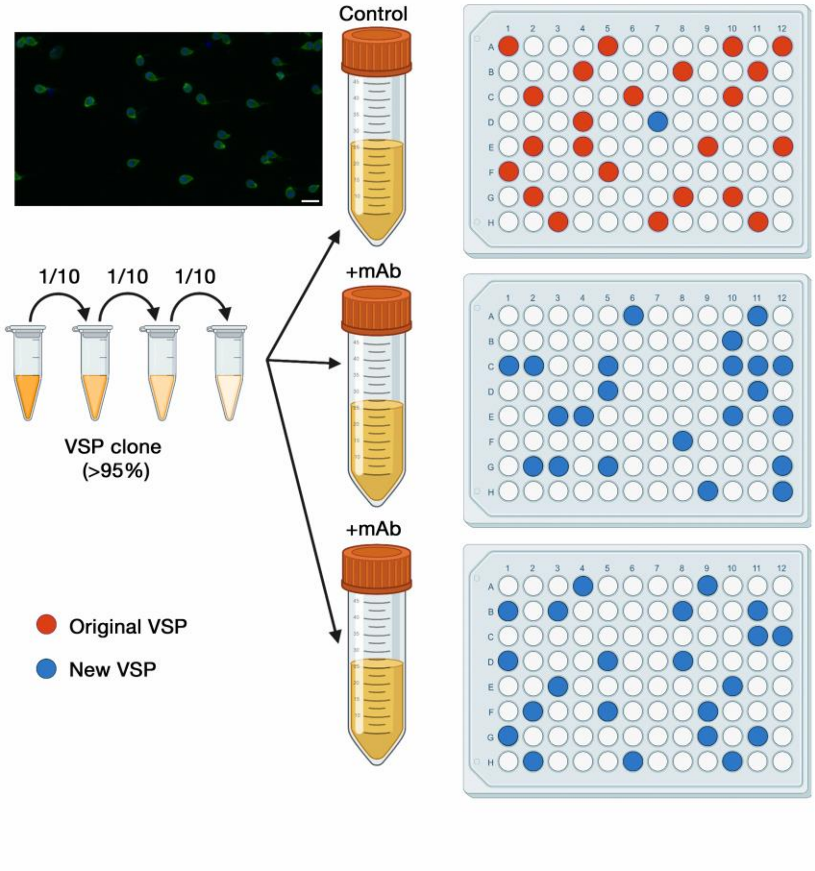

Expression of the original VSP	Original VSP / number of clones
Clone VSPH7 control	31/31
Clone VSP1267 control	27/28
Clone VSP417 control	27/28
Clone VSPH7 Exp. 1	1/27
Clone VSPH7 Exp. 2	0/29
Clone VSPH7 Exp. 3	1/31
Clone VSP1267 Exp. 1	1/33
Clone VSP1267 Exp. 2	0/35
Clone VSP1267 Exp. 3	0/30
Clone VSP417 Exp. 1	0/26
Clone VSP417 Exp. 2	1/37
Clone VSP417 Exp. 3	0/35

Supplementary Figure 1| Cytotoxicity and switching assay in *Giardia*. Left. Schematic representation of the *in-cell* antigenic switching assay (**left**). *Giardia* trophozoites expressing a particular VSP (almost 99 % positive by IFA; upper left corner) were subjected to limiting dilution to reach 50 trophozoites/ml and subsequently distributed in 96-well plates containing culture medium including either an unrelated mAb (mAb 8F12; top plate) or anti-VSP mAb (+mAb) at a final concentration of 50 nM. After 5 days of culture, the total number of growing clones and their reactivity to the mAb was determined. Orange wells represent clones expressing the original VSP and blue wells represent clones expressing a different VSP. **Right**, Table showing the number of clones expressing the original VSP vs. the total number of growing clones in three independent experiments. In the presence of the corresponding mAb, most clones expressed a different VSP after the treatment, demonstrating that anti-VSP antibodies induce antigenic variation. Scale bar 10 µm.

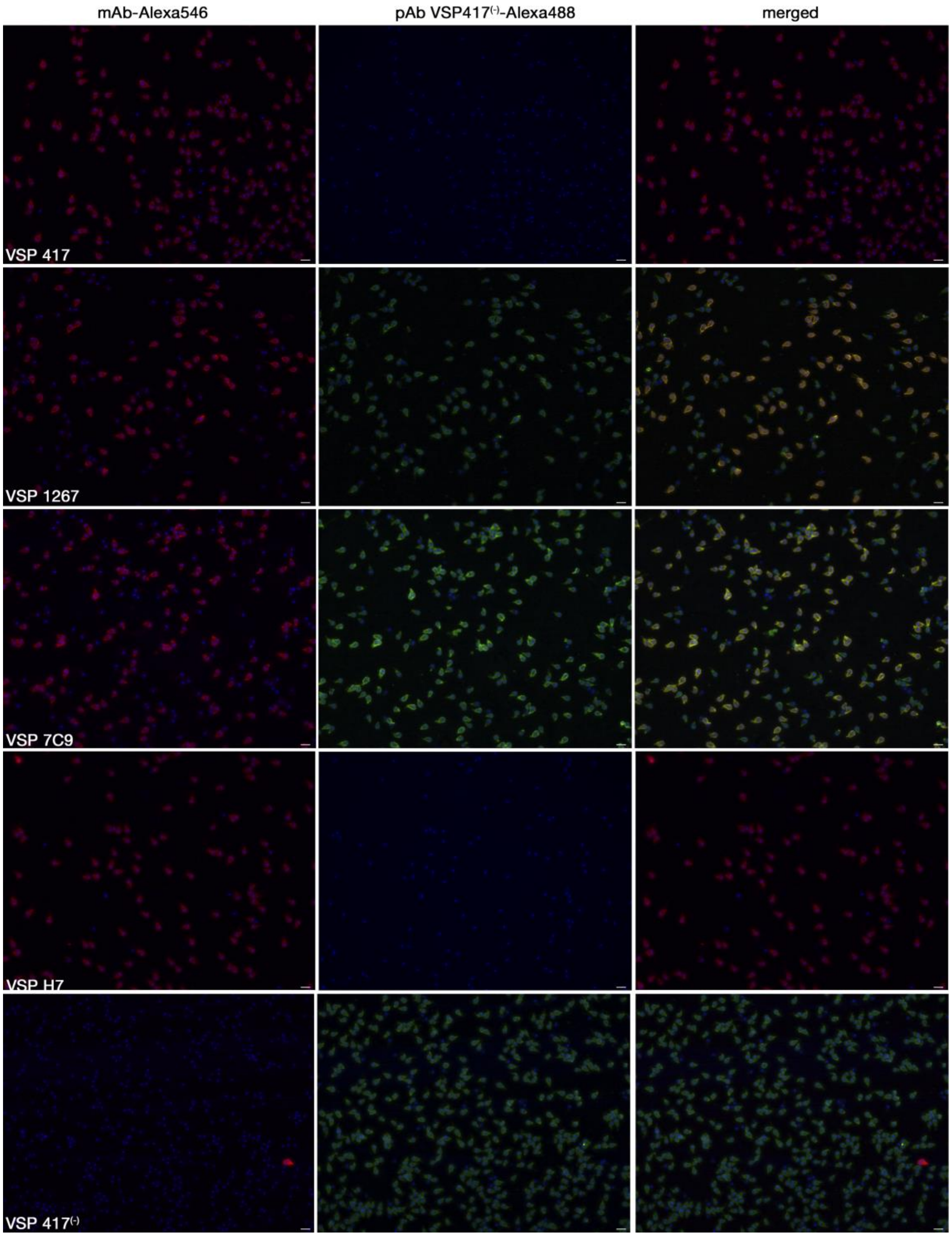

Supplementary Figure 2| Control of the specificity of the mouse polyclonal antibody VSP417$^{(-)}$. Trophozoites of clones expressing VSP417, VSP1267, VSP7C9, VSPH7 and a trophozoite population not expressing VSP417 were subjected to IFAs using their corresponding anti-VSP mAb labelled with Alexa Fluor-546 (red) and pAb VSP417$^{(-)}$ labelled with Alexa Fluor-488 (green). Nuclei were labelled with DAPI (blue). Overlays of the corresponding images are shown at the right. The pAb VSP417$^{(-)}$ could recognise VSPs other than VSP417 and VSPH7 (not present in the WB isolate). Scale bars 10 µm.

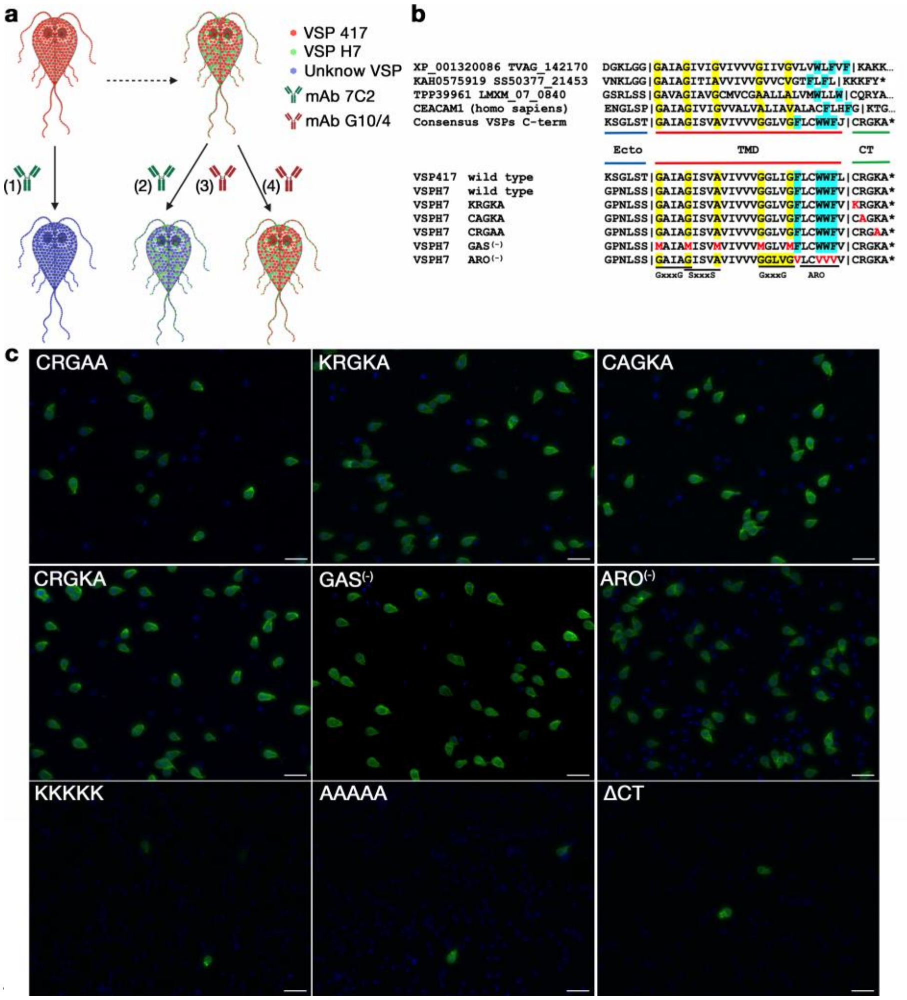

Supplementary Figure 3| The TMD of VSPs is involved in VSP clustering induced by anti-VSP antibodies in *Giardia*. a, Schematic representation of the experimental design for evaluating the involvement of the CT and the TMD of VSPs during antigenic switching. The diagram shows the reporter VSP417 in red (endogenously expressed) and the VSPH7 in green (constitutively expressed), the latter carrying different mutations in the C-terminal conserved region that includes the TMD and the CT (see b). Possibility 1 shows the induction of AV by antibodies to the endogenous VSP417. Possibilities 2-4 depict the different outcomes expected when parasites co-expressing two VSPs (VSP417 in red and VSPH7 in green) are exposed to either an anti-VSP417 or an anti-VSPH7 mAb. The induction of AV of the reporter VSP417 (option 3) or not (option 4), reflects the involvement or not of the conserved C-terminus of VSPs as a transducer of the antibody signal. **b**, Alignment of the C-terminal sequences of *Trichomonas vaginalis* G3 Hypothetical Protein XP_001320086 (V), *Spironucleus salmonicida* Cysteine-rich membrane protein 2 KAH0575919, *Leishmania Mexicana* Hypothetical protein CGC21_25575, human CEACAM1 and the consensus sequence of VSPs (top). Different variants of VSPH7 were designed to disrupt the GAS motifs (GAS$^{(-)}$) and the motif rich in aromatic amino acids (ARO$^{(-)}$). **c**, *In vivo* labelling of trophozoites using mAb G10/4 (left panel) showed surface localisation of each VSPH7 variant. Scale bars 10 µm.

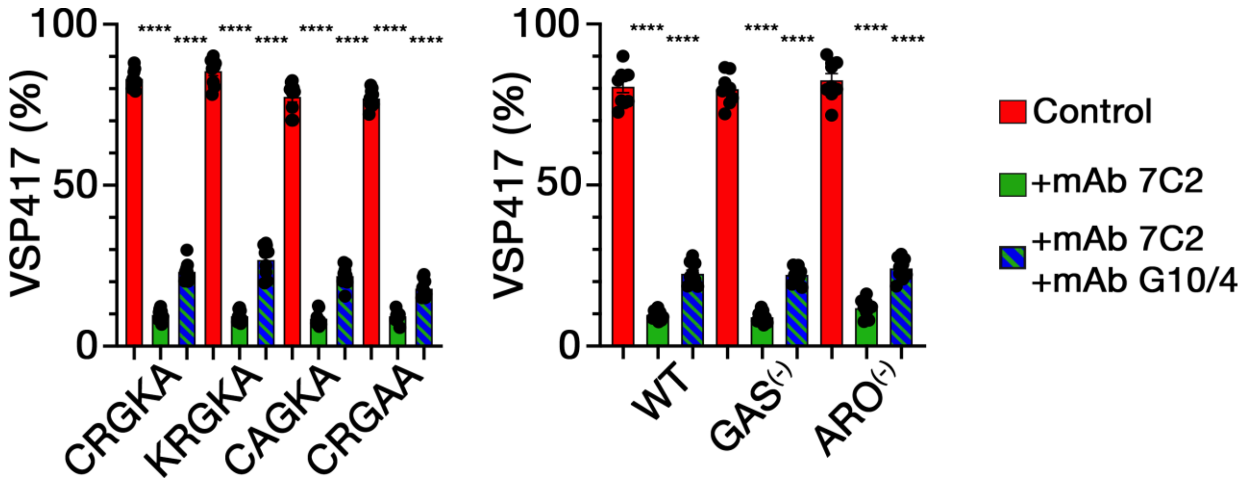

Supplementary Figure 4| Same experiment as in Fig. 2b and Fig. 2c but including treatments with a mixture of mAb 7C2 (anti-VSP417) and mAb G10/4 (anti-VSPH7) at 25 nM each (blue columns). Values represent mean ± s.e.m. of three independent experiments performed in triplicate. **p*<0.05; ***p*<0.01; ****p*<0.001. *****p*<0.0001; ns, not significant. Statistical significance is based on one-way ANOVA on datasets with Tukey's multiple comparisons tests.

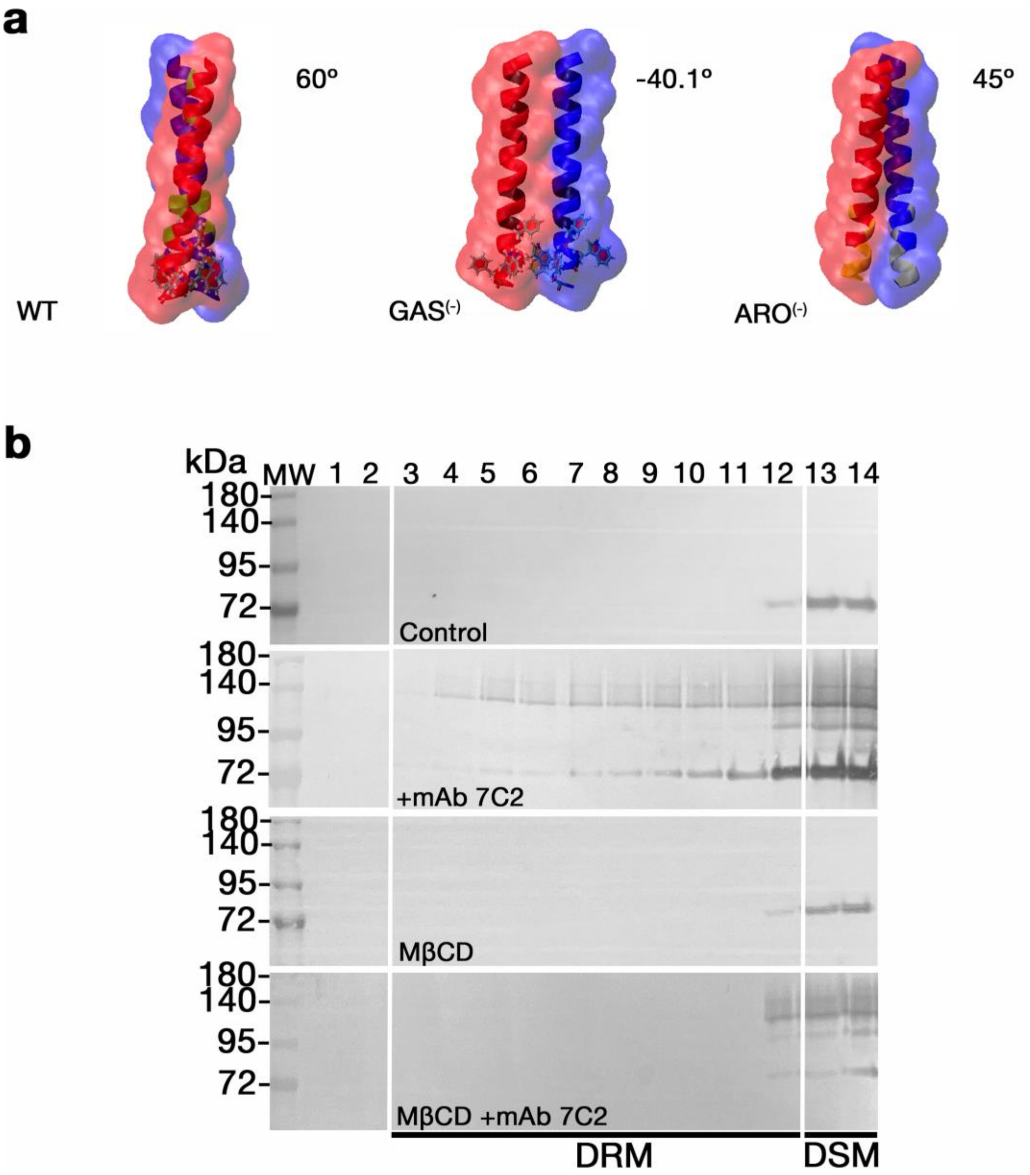

Supplementary Figure 5| The oligomerisation capability of the TMD of the VSP allows VSP redistribution into lipid rafts upon anti-VSP antibody binding. a, Modelling of the wild type (WT), GAS$^{(-)}$ and ARO$^{(-)}$ variants of the TMD of VSPH7 showing the differences in oligomerisation potential of the mutants compared to the WT TMD (Rosetta ddG: WT -51.94 > ARO$^{(-)}$ -35.59 > GAS$^{(-)}$ -21.86), **b**, Western blotting analysis of the distribution of VSP417 into detergent-resistant membranes (DRM) preparations compared to its presence in detergent soluble membranes (DSM) after treatment with 50 nM of mAb 7C2 (+mAb) or the unrelated mAb 8F12 for 1 h. The numbers on the top represent the fourteen collected fractions. Pre-treatment of the cells with methyl-β-cyclodextrin (10 mM) before incubation with mAb 7C2 or a control antibody is also shown (MβCD+mAb). Although at 1 h most of the VSP/antibody complex remains in the detergent soluble membrane (DRM) fractions, the complex redistributes into the detergent-resistant membrane (DRM) fractions upon treatment with the antibody. This redistribution is abolished if the trophozoites were previously treated with 10 mM of the cholesterol-rich disrupting domains MβCD.

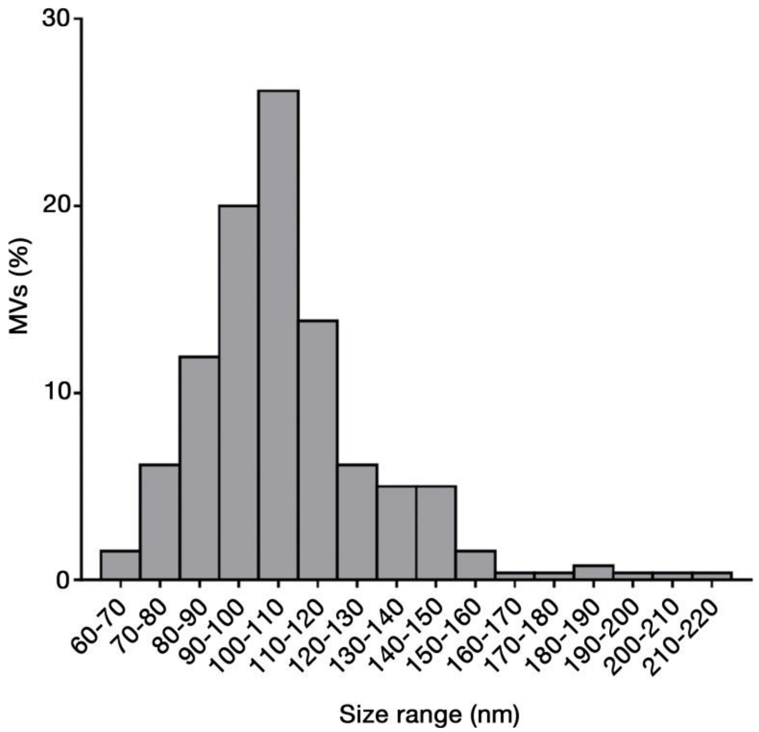

Supplementary Figure 6| Sizes of the released microvesicles in *Giardia*. Histogram showing the size distribution of the microvesicles as the percentage with respect to the total. Collected MVs from VSP417-expressing trophozoites exposed for 60 min to mAb 7C2 were resuspended in 100 µl of PBS and their size was determined by NanoSight analysis. Most of the released MVs range between 80 and 150 nm, with an average of ~100 nm.

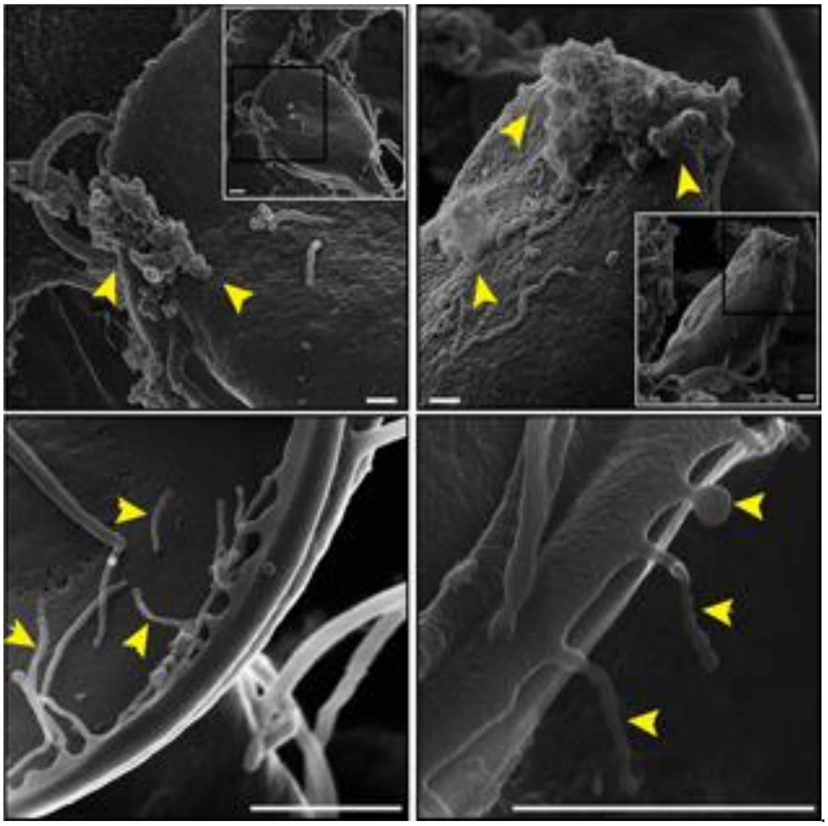

Supplementary Figure 7| VSPs are released into microvesicles. Additional extreme Resolution HI-SEM micrographs of VSP417 trophozoites recovered from the small intestine at 12 dpi. Extensive membrane projections and microvesicles emerging from the cell body and the ventral disc are shown (yellow arrows). Scale bars 1 µm.

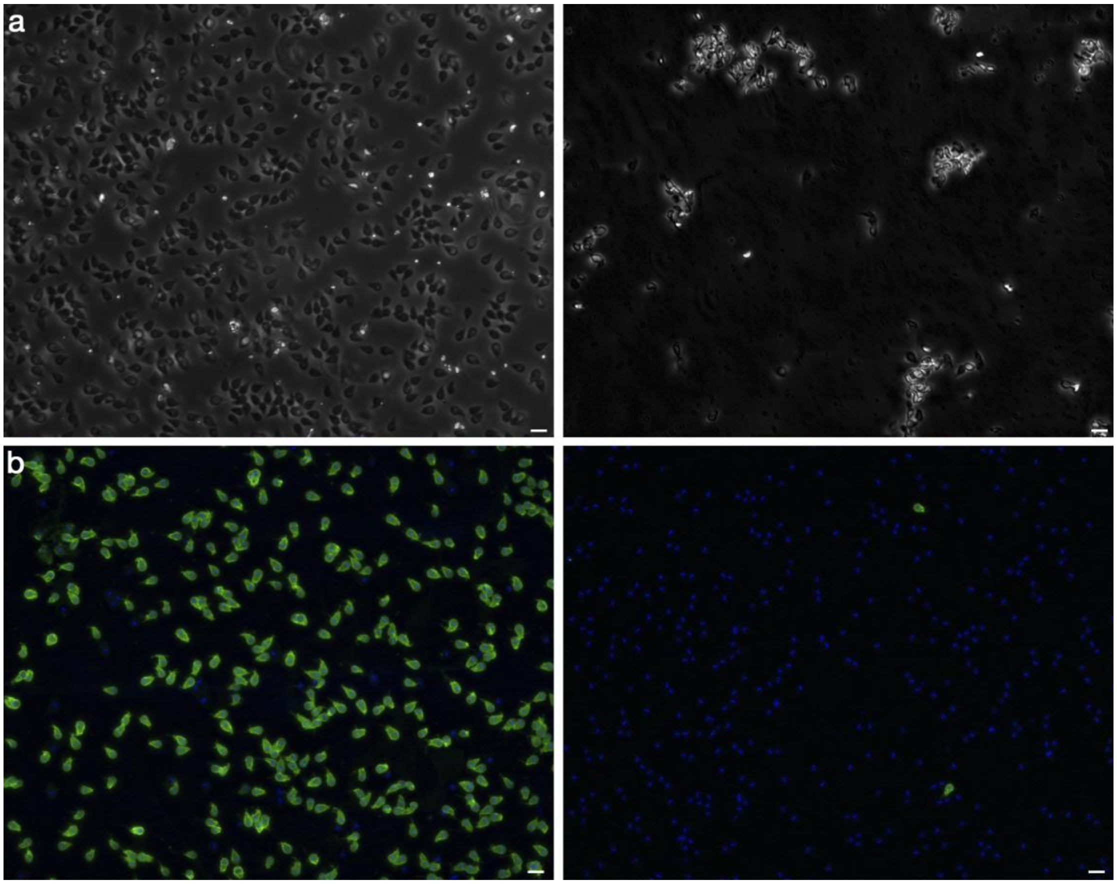

Supplementary Figure 8| Intestinal contents of gerbils infected with VSP417-expressing trophozoites induce antigenic variation *in vitro*. A. Phase contrast images of clone VSP417 incubated for 72 h with the intestinal contents of a not infected gerbil (left) or the intestinal content collected at day 12 post-infection of a gerbil infected with clone VSP417 (right). **B**. IFAs of *Giardia* trophozoites of clone expressing VSP417 using the anti-VSP417 mAb 7C2. Images in A show that the intestinal content of infected animals agglutinates the cells, in contrast to the control. Images in B show that the immunoglobulins present in the intestinal content at day 12 postinfection (right) induce switching in most of the trophozoites, suggesting that specific anti-VSP417 sIgA antibodies in the intestine of the infected animals also induce antigenic switching. Scale bars 10 µm.

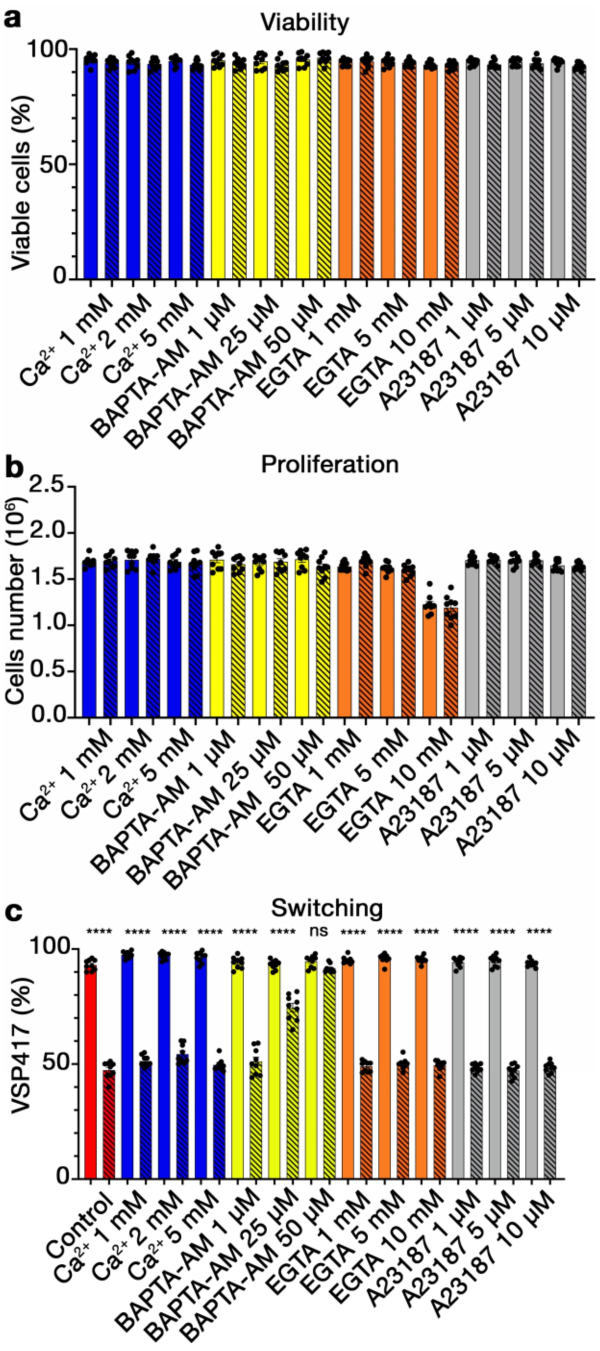

Supplementary Figure 9| a-c, **Effect of calcium on antibody-induced *Giardia* switching**. VSP417-expressing trophozoites were grown in the presence of a control mAb (solid bars) or mAb 7C2 (striped bars) for 72 h. The extracellular calcium concentrations, EGTA, the intracellular calcium chelator BAPTA-AM and the Ca^{2+} ionophore A23187 are shown. **a,** Viability. **b**, Proliferation. **c**, Switching. Values represent mean ± s.e.m. of three independent experiments performed in triplicate. *p<0.05; **p<0.01; ***p<0.001. ****p<0.0001; ns, not significant. Adjusted p-value= **c** BAPTA-AM 50μM = 0.8151 (ns). Statistical significance is based on one-way ANOVA on datasets with Tukey's multiple comparisons tests.
