# Supplementary material for: Antibodies to variable surface antigens induce antigenic variation in the intestinal parasite Giardia lamblia
Source: Nat Commun. 2023 May 3;14:2537. doi: 10.1038/s41467-023-38317-8 (PMC10156722; doi:10.1038/s41467-023-38317-8)
Supplement: Supplementary file 3 — Description of Additional Supplementary Information [file 41467_2023_38317_MOESM3_ESM.pdf]

Description of Additional Supplementary Information

Title: Supplementary movie 1

Description: Anti-VSP antibodies induce rapid detachment and agglutination of trophozoites without killing the parasites. Time-lapse observation of Giardia trophozoites expressing VSP417 during incubation with 100 µM of mAb 7C2. It can be seen how trophozoites quickly detach from the wall of the glass culture tubes and form large aggregates containing live cells. These large clumps of motile trophozoites remain unmodified for up to 3 days. (See separate .avi file)

Title: Supplementary data 1

Description: Proteomic analysis of purified microvesicles. Purified MVs obtained after 4 h of incubation with mAb 7C2 of trophozoites expressing VSP417 were subjected to proteomics analysis as described in Methods. Columns indicate the GenBank™ accession number, the locus tag, the original name of the proteins and a newly proposed one, the total number of identified peptides (SpC), the SpC of each independent experiment, the presence or absence of a TMD and a signal peptide (SP) and comment about the predicted subcellular localisations of all detected proteins are shown. VSPs are highlighted in yellow, α-giardins (annexins) in light blue and ALIX/PDCDIP in green. (See separate Excel table)
