# Supplementary material for: Antibodies to variable surface antigens induce antigenic variation in the intestinal parasite Giardia lamblia
Source: Nat Commun. 2023 May 3;14:2537. doi: 10.1038/s41467-023-38317-8 (PMC10156722; doi:10.1038/s41467-023-38317-8)
Supplement: Supplementary file 6 — Reporting Summary [file 41467_2023_38317_MOESM6_ESM.pdf]

nature portfolio

Corresponding author(s): HUGO D. LUJAN

Last updated by author(s): Apr 11, 2023

Reporting Summary

Nature Portfolio wishes to improve the reproducibility of the work that we publish. This form provides structure for consistency and transparency in reporting. For further information on Nature Portfolio policies, see our Editorial Policies and the Editorial Policy Checklist.

Statistics

For all statistical analyses, confirm that the following items are present in the figure legend, table legend, main text, or Methods section.

n/a	Confirmed	
☐	☒	The exact sample size (n) for each experimental group/condition, given as a discrete number and unit of measurement
☐	☒	A statement on whether measurements were taken from distinct samples or whether the same sample was measured repeatedly
☐	☒	The statistical test(s) used AND whether they are one- or two-sided<br>*Only common tests should be described solely by name; describe more complex techniques in the Methods section.*
☒	☐	A description of all covariates tested
☒	☐	A description of any assumptions or corrections, such as tests of normality and adjustment for multiple comparisons
☐	☒	A full description of the statistical parameters including central tendency (e.g. means) or other basic estimates (e.g. regression coefficient) AND variation (e.g. standard deviation) or associated estimates of uncertainty (e.g. confidence intervals)
☐	☒	For null hypothesis testing, the test statistic (e.g. F, t, r) with confidence intervals, effect sizes, degrees of freedom and P value noted<br>*Give P values as exact values whenever suitable.*
☒	☐	For Bayesian analysis, information on the choice of priors and Markov chain Monte Carlo settings
☒	☐	For hierarchical and complex designs, identification of the appropriate level for tests and full reporting of outcomes
☒	☐	Estimates of effect sizes (e.g. Cohen's d, Pearson's r), indicating how they were calculated

Our web collection on statistics for biologists contains articles on many of the points above.

Software and code

Policy information about availability of computer code

Data collection	NCBI Giardia lamblia RefSeq 2.1
Data analysis	FlowJoTM 7.6 (TreeStar).<br>HCImage software U11158.<br>ImageJ 1.49 software.<br>ZEN 3.6 software (Zeiss).<br>GraphPad 8.2.1 (279) (Prism).<br>Mascot 2.8.2 (Matrix Science)

For manuscripts utilizing custom algorithms or software that are central to the research but not yet described in published literature, software must be made available to editors and reviewers. We strongly encourage code deposition in a community repository (e.g. GitHub). See the Nature Portfolio guidelines for submitting code & software for further information.

Data

Policy information about availability of data

All manuscripts must include a data availability statement. This statement should provide the following information, where applicable:

- Accession codes, unique identifiers, or web links for publicly available datasets
- A description of any restrictions on data availability
- For clinical datasets or third party data, please ensure that the statement adheres to our policy

Source data are provided in this paper. All other data are available in the main text or the supplementary materials. Proprietary antibodies are available upon request through a material transfer agreement (MTA). The mass spectrometry proteomics data have been deposited at the ProteomeXchange Consortium via the PRIDE partner repository with the dataset identifiers PXD031141 and 10.6019/PXD031141.

Human research participants

Policy information about studies involving human research participants and Sex and Gender in Research.

Reporting on sex and gender	N/A
Population characteristics	N/A
Recruitment	N/A
Ethics oversight	N/A

Note that full information on the approval of the study protocol must also be provided in the manuscript.

Field-specific reporting

Please select the one below that is the best fit for your research. If you are not sure, read the appropriate sections before making your selection.

☒ Life sciences ☐ Behavioural & social sciences ☐ Ecological, evolutionary & environmental sciences

For a reference copy of the document with all sections, see nature.com/documents/nr-reporting-summary-flat.pdf

Life sciences study design

All studies must disclose on these points even when the disclosure is negative.

Sample size	In general, sample sizes were of an n of 9 for in vitro experiments.
Data exclusions	No data were excluded.
Replication	Experiments were performed in triplicate and validated independently.
Randomization	Randomization was used.
Blinding	All experiments in vitro were blinded and performed by three independent researchers. Experiments in animal were not blinded.

Reporting for specific materials, systems and methods

We require information from authors about some types of materials, experimental systems and methods used in many studies. Here, indicate whether each material, system or method listed is relevant to your study. If you are not sure if a list item applies to your research, read the appropriate section before selecting a response.

Materials & experimental systems

n/a	Involved in the study
☐	☒ Antibodies
☒	☐ Eukaryotic cell lines
☒	☐ Palaeontology and archaeology
☐	☒ Animals and other organisms
☒	☐ Clinical data
☒	☐ Dual use research of concern

Methods

n/a	Involved in the study
☒	☐ ChIP-seq
☐	☒ Flow cytometry
☒	☐ MRI-based neuroimaging

Antibodies

Antibodies used

5C1 IgM VSP1267/GL50803_00112208/XP_001706567.1 A1 WB
7F5 IgG1 VSP1267/GL50803_00112208/XP_001706567.1 A1 WB
7C2 IgG1 VSP417/GL50803_00113797/XP_001710078.1 A1 WB
9B10 IgG1 VSP9B10/GL50803_00101074/XP_001706983.1 A1 WB
2B10 IgM VSPAS8/GL50803_0040591/XP_001708393.2 A1 WB
7C9 IgM VSP7C9/GL50803_0050375/XP_037901545.1 A1 WB
6E7 IgG1 VSPA6/GL50803_00221693/XP_001707734.2 A1 WB
G10/4 IgG1 VSPH7/GSB_150963/ESU41081.1 B GS
8F12 IgG2a CWP1/GL50803_005638/XP_001704890.1 A1 WB
VSP417(-) pAb A1 WB
goat anti-mouse IgG-(H-L)-gold 10 nm (Abcam, Cat. # ab39619)
goat anti-mouse IgG (H-L)-PE (InvitrogenTM, Cat. # PA1-84395)
goat anti-rat IgG (H-L)-biotin 1/2000 (InvitrogenTM, Cat. # A18869)
streptavidin-Alexa FluorTM 488 (InvitrogenTM, Cat. # S11223)
goat anti-mouse IgG (H+L)-Alexa FluorTM 488 (InvitrogenTM, Cat. # A11001)
goat anti-rat IgG (H-L)-Alexa FluorTM 546 (InvitrogenTM, Cat. # A11081)
peroxidase-conjugated goat anti-mouse IgG (H-L) (InvitrogenTM, Cat. # 626520)

Validation

All propietary antibodies were validated previously by Western Blot and Immunofluorescence assays as reported in:
Serradell MC, Rupil LL, Martino RA, et al. Efficient oral vaccination by bioengineering virus-like particles with protozoan surface proteins. Nat Commun. 2019;10(1):361. Published 2019 Jan 21. doi:10.1038/s41467-018-08265-9
Rivero FD, Saura A, Prucca CG, Carranza PG, Torri A, Lujan HD. Disruption of antigenic variation is crucial for effective parasite vaccine. Nat Med. 2010;16(5):551-557. doi:10.1038/nm.2141
Prucca CG, Slavin I, Quiroga R, et al. Antigenic variation in Giardia lamblia is regulated by RNA interference. Nature. 2008;456(7223):750-754. doi:10.1038/nature07585
Luján HD, Mowatt MR, Conrad JT, Bowers B, Nash TE. Identification of a novel Giardia lamblia cyst wall protein with leucine-rich repeats. Implications for secretory granule formation and protein assembly into the cyst wall. J Biol Chem. 1995;270(49):29307-29313. doi:10.1074/jbc.270.49.29307

Animals and other research organisms

Policy information about studies involving animals; ARRIVE guidelines recommended for reporting animal research, and Sex and Gender in Research

Laboratory animals

Meriones unguiculatus (gerbils) (6-8 week-old) were used for infection experiments, Balb/c mice (6-8 week-old) for the generation of monoclonal antibodies and Wistar rats (6-8 week-old) for the production of polyclonal antibodies. All animals were obtained from the vivarium of the CIDIE and used as described in Methods.

Wild animals

Wild animals were not used

Reporting on sex

BALB/c mice, Wistar rats and gerbils of both sexes were used

Field-collected samples

N/A

Ethics oversight

CICUAL protocols CIDIE.2016-36-15p-2, and CIDIE.2018-36-15p-3. CIDIE: Centro de Investigación y Desarrollo en Inmunología y Enfermedades Infecciosas.

Note that full information on the approval of the study protocol must also be provided in the manuscript.

Flow Cytometry

Plots

Confirm that:

☒ The axis labels state the marker and fluorochrome used (e.g. CD4-FITC).

☒ The axis scales are clearly visible. Include numbers along axes only for bottom left plot of group (a 'group' is an analysis of identical markers).

☒ All plots are contour plots with outliers or pseudocolor plots.

☒ A numerical value for number of cells or percentage (with statistics) is provided.

Methodology

Sample preparation	Giardia trophozoites expressing diferent VSP were confronted to their particula anti VSP mAb.
Instrument	BD Accuri C6.
Software	FlowJoTM 7.6 (TreeStar)
Cell population abundance	Density plots were used reflecting the percentage of any given population.
Gating strategy	Compensation was set to account for spectral overlap between the two fluorescent channels. No gating strategy was used but regions were set as quadrants reflecting positive staining for VSP417, positive staining for VSPs other than VSP417, and positive for both dyes. Density plots were displayed, indicating the percentage of each population.

☐ Tick this box to confirm that a figure exemplifying the gating strategy is provided in the Supplementary Information.
